# Adaptive Evolution Coupled with Retrotransposon Exaptation Allowed for the Generation of a Human-Protein-Specific Coding Gene That Promotes Cancer Cell Proliferation and Metastasis in Both Haematological Malignancies and Solid Tumours: The Extraordinary Case of *MYEOV* Gene

**DOI:** 10.1155/2015/984706

**Published:** 2015-10-19

**Authors:** Spyros I. Papamichos, Dimitrios Margaritis, Ioannis Kotsianidis

**Affiliations:** Department of Haematology, School of Medicine, Democritus University of Thrace, 68100 Alexandroupolis, Greece

## Abstract

The incidence of cancer in human is high as compared to chimpanzee. However previous analysis has documented that numerous human cancer-related genes are highly conserved in chimpanzee. Till date whether human genome includes species-specific cancer-related genes that could potentially contribute to a higher cancer susceptibility remains obscure. This study focuses on *MYEOV*, an oncogene encoding for two protein isoforms, reported as causally involved in promoting cancer cell proliferation and metastasis in both haematological malignancies and solid tumours. First we document, via stringent *in silico* analysis, that *MYEOV* arose *de novo* in Catarrhini. We show that MYEOV short-isoform start codon was evolutionarily acquired after Catarrhini/Platyrrhini divergence. Throughout the course of Catarrhini evolution *MYEOV* acquired a gradually elongated translatable open reading frame (ORF), a gradually shortened translation-regulatory upstream ORF, and alternatively spliced mRNA variants. A point mutation introduced in human allowed for the acquisition of MYEOV long-isoform start codon. Second, we demonstrate the precious impact of exonized transposable elements on the creation of *MYEOV* gene structure. Third, we highlight that the initial part of MYEOV long-isoform coding DNA sequence was under positive selection pressure during Catarrhini evolution. *MYEOV* represents a Primate Orphan Gene that acquired, via ORF expansion, a human-protein-specific coding potential.

## 1. Introduction

It has formerly been suggested that at least some of human (*Homo sapiens*) major diseases could partially likely be related to genetic maladaptations during the recent evolutionary past [1]. The incidence of cancer in human is rather high as compared to chimpanzee (*Pan troglodytes*) [2]. However, previous analysis [3] has shown that numerous human cancer-related genes are highly conserved in chimpanzee, containing intact open reading frames (ORFs). Only minor differences were reported between species [3]. Therefore whether human genome includes species-specific cancer-related genes or gene isoforms that could potentially contribute to a higher cancer susceptibility remains yet obscure.

Of note,* MYEOV* (also known as* OCIM*, National Center for Biotechnology Information [NCBI] Gene ID: 26579) was not included in the human cancer gene data set used in the analysis by Puente et al. [3].* MYEOV* is a noncensus cancer gene that during the last 15 years has been reported as causally involved in promoting cancer cell proliferation and metastasis in both haematological malignancies and solid tumours [4–11].

The gene has the potential to generate via alternative splicing six mRNA variants encoding for two protein isoforms [6, 9, 12], namely, for a 313-amino-acid (aa) peptide (MYEOV-313) as well as for a shorter 255-aa peptide (MYEOV-255). Solid Western-blot assays support the production of both the proteins [9, 12] while MYEOV-313 expression has been associated with poor prognosis in patients with multiple myeloma [9]. Both MYEOV-313 and MYEOV-255 seem to be directed to the membrane [6] but are of, yet, unknown biological function.

*MYEOV* has been shown to be epigenetically regulated via a DNA-methylation mechanism [5] while at the protein synthesis level the gene seems to be tightly controlled by upstream ORFs (uORFs) [12]. Importantly,* MYEOV* second pseudosignal generates a long, weak Kozak signal, uORF that could significantly impair translational efficiency [13–15].

In 2012,* MYEOV* was characterized as a “*Class I*” hominoid-specific* de novo* protein-coding gene [16],* id est*, deduced to encode for protein exclusively in human. Xie et al. [16] identified an ORF disrupting mutation that was present in* MYEOV* syntenic locus in multiple higher primates, ranging from chimpanzee to rhesus monkey (*Macaca mulatta*). Intriguingly, the “shared disabler” [17] located in MYEOV-313 start codon [16]. Accordingly, the authors [16] excluded* MYEOV* origination via other molecular mechanisms known to generate novel genes [18] and annotated* MYEOV* as a human-specific* de novo* protein-coding gene, eventually concluding that it is likely* de novo* origination rather than ORF expansion that drove the origination of hominoid-specific genes [16], such as* MYEOV*.

Transposable elements (TEs) are known to exert a broad evolutionary impact, promoting genomic plasticity and eventually biological diversity [19, 20]. Exonized TEs have the potential to induce significant changes in functional noncoding regions of genes while, in extraordinary cases, some could even acquire a novel role as protein-coding modules [21–24], in a biological process called “exaptation” [25]. Interestingly, it was relatively recently documented that TEs could preciously contribute in* de novo* gene-origination [26–29].

In this paper, we argue about* MYEOV* representing a human-specific* de novo* protein-coding gene. We show, via* in silico* analysis, that* MYEOV* arose* de novo* in Catarrhini and that it was adaptive evolution coupled with TE exaptation that eventually allowed for the generation of a human-specific primary ORF.

## 2. Materials and Methods

### 2.1. Inquiring into* MYEOV* Origin and Validating* MYEOV* Orthologous Sequences

*MYEOV* locus syntenic alignments of numerous vertebrates were extracted from the “Multiz Alignments of 100 Vertebrates” track of the UCSC Genome Browser Database [30–33].

BLASTN [34] search against the GenBank human genomic database and BLASTP [34] search against the GenBank nonredundant protein database were used to exclude* MYEOV* origination via gene duplication and to verify unambiguous* MYEOV* orthologs; the stringent filters described previously were applied [16, 35].

*MYEOV* orthologs annotated in the respective databases NCBI, Ensembl, and OrthoDB [36] were identified by searching with the gene name. Whole-genome shotgun (WGS) sequence contigs, including within the DNA segments used to curate the reference genomic sequences of* MYEOV* orthologs, were downloaded from the NCBI Gene database (http://www.ncbi.nlm.nih.gov/gene/). The WGS sequence contigs were used to build* MYEOV* locus alignment blocks between human and the species with annotated orthologs, via Clustal Omega [37]. Each alignment block was manually scrutinized for validating the primary ORF (pORF) of the annotated orthologs.

To estimate the degree of evolutionary constraint on* MYEOV* coding DNA sequence during evolution in the Catarrhini genome, the average human-rhesus *K*_*a*_/*K*_*s*_ ratio was calculated [38]; MEGA6 software was used in the analysis [39]. Ratios for* MYEOV* second and third exon coding DNA sequences were calculated separately.

Provided in the study by Finstermeier et al. [40] is a beautiful phylogenetic tree that clarifies the nomenclature and supports the mammalian classification and the estimated divergence ages used in the text.

### 2.2. Scanning* MYEOV* and* MYEOV* Evolutionary Antecedents for the Presence of Integrated TEs

*MYEOV* and* MYEOV* orthologous genomic sequences were scanned for the presence of TEs by RepeatMasker [41]. The program was run in both default and sensitive mode, using a matrix optimal for* MYEOV* GC level to avoid false masking. Respective searches using PLOTREP [42], the TranspoGene database [43], and BLASTN [34, 44] were also performed.

*MYEOV* locus automated alignments downloaded from the UCSC Genome Browser Database [30–33] were used to extract syntenic DNA segments in numerous mammals that flank, 500 nucleotides (nts) in the 5′ and the 3′, MYEOV-313 start codon. BLASTN was used to align these DNA segments with corresponding WGS sequence contigs for extracting the original DNA sequence in each species. That is because the “Multiz Alignments of 100 Vertebrates” track of the UCSC Genome Browser Database excludes multiple inserts located between the alignment blocks of the synteny. Each WGS sequence contig was then realigned to human* MYEOV*. The DNA segments eventually extracted were scanned by RepeatMasker [41] for the presence of integrated TEs.

### 2.3. *MYEOV* Upstream ORF and Splice Site Computational Analysis

Search for* MYEOV* uORFs was performed with the ORF Finder program (http://www.bioinformatics.org/sms2/orf_find.html). Search parameters were set as previously suggested [45].

*MYEOV* core splicing signal strength values were accessed with the Human Splicing Finder Version 2.4.1 program [46], as previously described [47].

### 2.4. Searching for mRNA Expression Evidence in Nonhuman Primates

BLASTN searches with human* MYEOV* mRNA sequences against the GenBank expressed sequence tags (EST) database were used to identify matching expressed mRNA sequences in nonhuman primates.

### 2.5. *MYEOV* Common Single Nucleotide Polymorphisms and Confirmed Somatic Mutations Analysis

Common single nucleotide polymorphisms (SNPs), located in* MYEOV* genomic sequence, were extracted from the “Common SNPs” track of the UCSC Genome Browser Database [30–33].

*MYEOV* confirmed somatic mutations annotated in the catalogue of somatic mutations in cancer (COSMIC) [48] were identified by searching with the gene name.

## 3. Results and Discussion

### 3.1. From a Eutherian-Mammal Noncoding Sequence to a Human-Protein-Specific Coding Gene

*MYEOV* BLASTN search against the GenBank human genomic database yields no significant similarity with any coding genomic sequences other than itself.

Syntenic alignments, extracted from the “Multiz Alignments of 100 Vertebrates” track of the UCSC Genome Browser Database [30–33], signify that the DNA segment where* MYEOV* locates emerged in eutherian mammals. The DNA segment is absent in all outgroups ranging from marsupials to lamprey. Of note a number of species, for example, Myomorpha, Sciuromorpha, and Lagomorpha, lack a large portion of the syntenic region (Supplementary Figure 1 in Supplementary Material available online at http://dx.doi.org/10.1155/2015/984706). This finding could explicate the inability to detect a mouse* myeov* transcript via zoo blot analyses under low-stringency hybridization conditions, as reported previously [6].

*MYEOV* is reported in the Gene database from NCBI (http://www.ncbi.nlm.nih.gov/gene/) to have sixteen orthologous genes, exclusively present in higher primates (Anthropoidea). Fifteen protein-coding genes as well as a pseudo-ortholog in rhesus monkey are annotated.

MYEOV-313 BLASTP search against the GenBank nonredundant protein database [34] yields however an intriguing finding. The peptide predicted to be encoded from Bolivian squirrel monkey (*Saimiri boliviensis*)* MYEOV* ortholog presents higher amino acid coverage with MYEOV-313 protein compared to the respective peptide encoded from chimpanzee* MYEOV* ortholog, despite Bolivian squirrel monkey being a primate far more distantly related to human than chimpanzee is.

Of note, OrthoDB [36] reports yet another* MYEOV* protein-coding ortholog in dolphin (*Tursiops truncatus*), raising the issue of the locus functionality in ancestral genomes to be subsequently lost in multiple lineages and later regained in higher primates [17, 49, 50].

The plausible interpretation of these intriguing findings is that some of the automated annotations of* MYEOV* orthologous ORFs provided by the NCBI Eukaryotic Genome Annotation Pipeline and the Ensembl Genebuild [51] contain inaccuracies [49]. Elucidated below is the evolutionary path leading to* MYEOV*.

**Figure 1:**
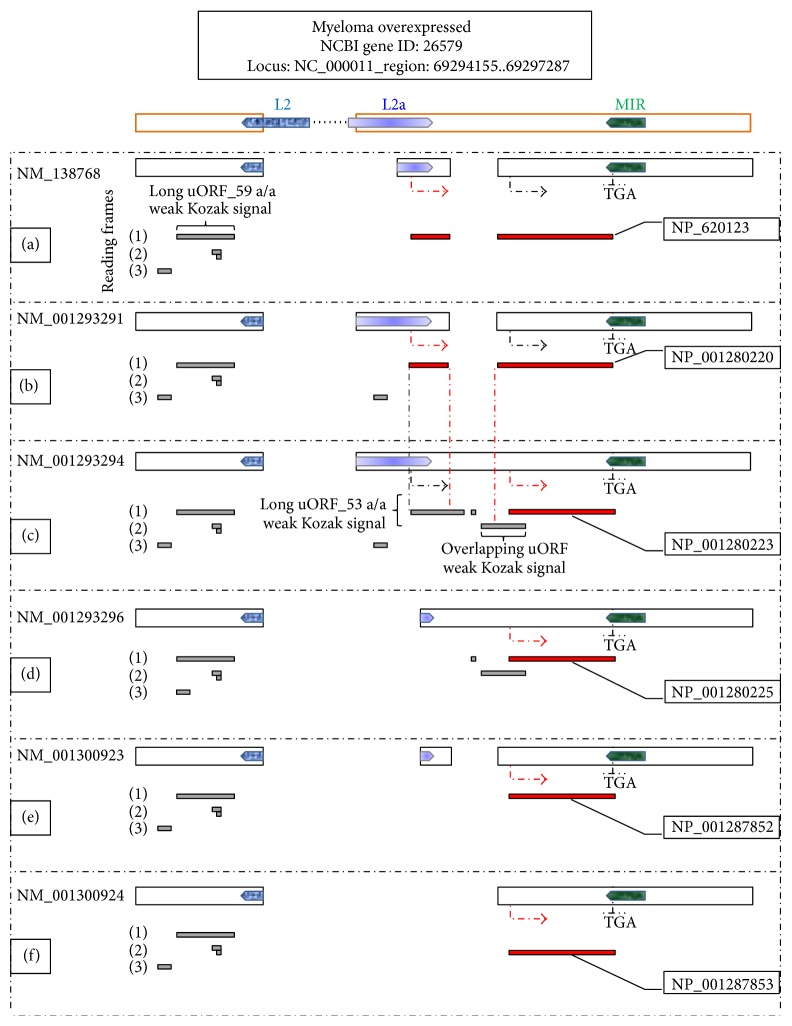
*MYEOV* alternatively spliced variants. Illustrated on top is the genomic organization of* MYEOV* locus. Bold, orange line boxes correspond to exonic segments of the genomic sequence. Pentagons overlapping the boxes delineate the location and orientation of the three TEs referred to in the text. (a–f) Six* MYEOV* alternatively spliced variants encode for either MYEOV-313 or MYEOV-255, according to the NCBI Gene database. Bold, black line boxes for* MYEOV* exonic content corresponding to each variant. Directional arrows correspond to the functional start signals of* MYEOV*; red directional arrows are for the start codons demarcating the respective pORF in each variant. The common stop codon location (TGA) is also indicated. Provided below the exonic content, depicted as dark-grey horizontal bars and juxtaposed to the respective pORFs (red horizontal bars), are the uORFs present in each mRNA sequence; long uORFs are annotated in the context of significantly impairing translational efficiency. Numbers in parentheses correspond to consecutive reading frames, starting from the first, second, and third nucleotide of each variant. Importantly, a prerequisite for* MYEOV* long-ORF to occur is the region demarcated by red vertical lines to be spliced out of the mRNA sequence.

**Figure 2:**
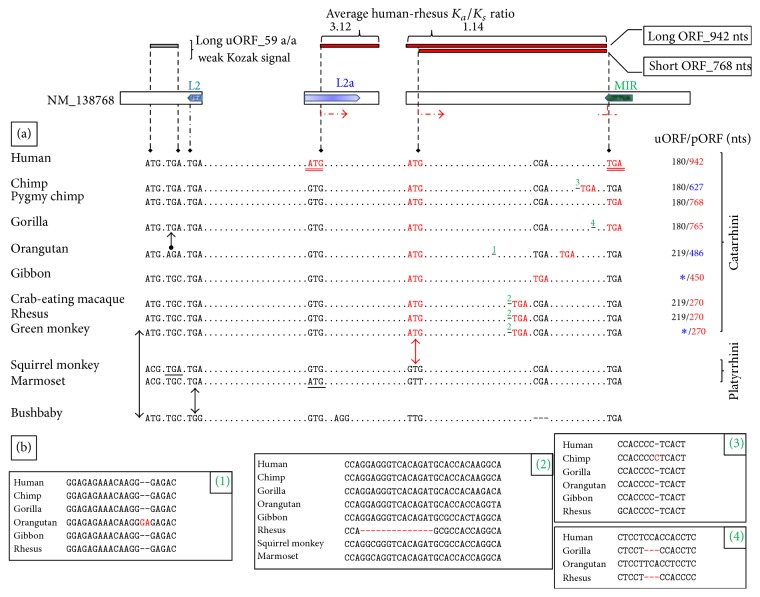
From a noncoding sequence to* MYEOV*. Depicted is* MYEOV* transcript variant 2 (NM_138768) as described in Figure 1; illustrated are both the translatable ORFs (red horizontal bars) and the long uORF (dark-grey horizontal bar) present in the variant. Shown also are the average human-rhesus *K*_*a*_/*K*_*s*_ ratios calculated separately for* MYEOV* second and third exon coding DNA sequences. (a) Black, discontinuous, vertical lines point to precious trinucleotides of* MYEOV* sequence, referred to within the text. Appearing underlined, in red font, are the trinucleotides that demarcate MYEOV-313 coding sequence. Aligned below the human sequence are corresponding trinucleotides from* MYEOV* syntenic region in 11 primates.* Bona fide* pORFs in* MYEOV* orthologs are delimited by respective red font trinucleotides; out-of-frame stop codons are presented unaligned. Double-headed red arrow points to the* de novo* acquisition of MYEOV-255 start codon in Catarrhini. MYEOV-313 start signal is human-specific; an identical* ATG* trinucleotide appearing in marmoset syntenic region (underlined) is due to a parallel mutation. Double-headed black arrows are for the trinucleotides demarcating the primal 219 nts long uORF that arose in Catarrhini. A black directional arrow points out the shortening of this long uORF in Hominines; an identical* TGA* trinucleotide occurring in squirrel monkey syntenic region (underlined) is likely due to a parallel mutation. Shown in the right flank is the size of the long uORF/pORF corresponding to each species. The sizes of the pORFs present in chimpanzee and Sumatran orangutan* MYEOV* orthologs (appearing in blue font) require decidedly additional validation due to the presence of ORF-shifting indels in the respective sequences. Blue asterisks for gibbon- and green monkey-specific indels that transmute the respective uORFs. Alignment gaps in bushbaby syntenic region were inserted for clarity. (b) Provided within boxes, numbered in accordance with the superscripts numbers appearing in (a), are syntenic alignments among various higher primates including the indels referred to in the text. With regard to the Cercopithecoidea-specific 14 bp deletion, shown is only one of these species due to space limitations.

Interestingly, one of the first functional genetic modules that emerged during* MYEOV* evolution in the mammalian genome was the long uORF, reported previously to participate in the regulation of the gene's translational efficiency [12] (Figure 1). The upstream* ATG* trinucleotide demarcating this long uORF seems fixed in Catarrhini but in depth phylogenetic analysis indicates that it could be of ancestral status (Figure 2). The trinucleotide appears transmuted in Platyrrhini probably due to either emergence of the* ACG* trinucleotide in a common Platyrrhini ancestor or parallel mutations in these species. With regard to the* TGA* trinucleotide originally delimiting the long uORF, it appears evolutionarily fixed in higher primates. Eventually the presence of these two trinucleotides in the genomic sequence allowed for the occurrence in Catarrhini of a primal 219 nts long uORF (Figure 2).

*Circa* (*ca.*) 46 Ma (Catarrhini/Platyrrhini divergence time) [40], an important mutational event occurred in Catarrhini resulting in the* de novo* gain of a potentially functional ORF in a genomic region that is noncoding in other primates; that is the acquisition of MYEOV-255 start codon (Figure 2).

*Ca.* 15.2 Ma (Homininae/Ponginae divergence time) [40] another precious point mutation allowed in Hominines for the shortening of* MYEOV* long uORF (Figure 2), an event rather meaningful in the context of the gene's translational regulation [45]. Of note, an identical* TGA* trinucleotide occurring in Bolivian squirrel monkey syntenic region is likely due to a parallel mutation (Figure 2).

Finally,* ca.* 5.9 Ma (*Homo*/*Pan* separation time) [40] a momentous point mutation was introduced in human resulting in the acquisition of MYEOV-313 start codon (Figure 2). An identical trinucleotide appearing in white-tufted-ear marmoset (*Callithrix jacchus*) syntenic region is due to a parallel mutation and is likely nonfunctional.

Notably, the stop codon delimiting the relatively short pORFs in Old World monkeys (Cercopithecoidea) represents not an ancestral frame disrupting feature that the corresponding hominoid orthologs escaped from since this trinucleotide occurs exclusively in Old World monkeys (Supplementary Data Set 1). In addition it is off-frame with regard to* MYEOV* coding sequence (Figure 2, Supplementary Data Set 1). That is due to a 14-bp deletion, also fixed exclusively in Old World monkeys, locating short upstream from the stop codon (Figure 2). In the same sense, the stop codon demarcating the pORF in gibbon (*Nomascus leucogenys*) occurs exclusively in gibbon and orangutan (*Pongo abelii*). It would feel somewhat more plausible to speculate that parallel mutational events took place in both species rather than assuming the occurrence of a point mutation in a common hominoid ancestor, suffering refutation and evolutionary “refixation” to the antecedent state in succeeding lineages (Figure 2). Of note an orangutan-specific insertion, requiring decidedly additional validation due to shifting very early the respective orangutan* MYEOV* pORF, translocates out-of-frame this* TGA* trinucleotide (Figure 2, Supplementary Data Set 1).

All the above could signify that* MYEOV* third exon coding DNA sequence (Figure 2) was not under strong selective constraints during evolution in the Catarrhini genome [38, 52]. Reinforcing the above, this sequence yields an average human-rhesus *K*_*a*_/*K*_*s*_ ratio of 1.14. Accordingly, it was likely neutral drift-directed evolution [53] that drove the 3′ expansion of* MYEOV* pORF (Figure 2).

On the other hand,* MYEOV* second exon coding DNA sequence yields an average human-rhesus *K*_*a*_/*K*_*s*_ ratio of 3.12. Thus the 5′ expansion of* MYEOV* pORF during the gene's evolution in the Catarrhini genome, including the acquisition of the human-specific start codon, was likely driven under positive selection pressure [54] (Figure 2).

### 3.2. Precious Exapted TEs Present in* MYEOV* Genomic Sequence

*MYEOV* scan by RepeatMasker reveals the presence of seven TEs in the genomic sequence (Supplementary Data Set 2). Importantly, nonredundant* MYEOV* modules seem to localize within three of these genetic elements.

In detail, the constitutively used donor of* MYEOV* first splicing junction locates in a region reported from RepeatMasker to match an antisense orientation L2 repeat of the long interspersed nuclear element (LINE) class (Figure 1). Of note, this TE provided also the stop codon delimiting* MYEOV* primal 219 nts long uORF, discussed above (Figure 2).

The stop codon that is common to both MYEOV-313 and MYEOV-255 protein isoforms locates in a region reported from RepeatMasker to match an antisense orientation mammalian-wide interspersed repeat (MIR) of the short interspersed nuclear element (SINE) class (Figure 1).

Most importantly the three alternative acceptors of* MYEOV* first splicing junction and the initial region of MYEOV-313 coding sequence, including the start codon, are located in a region reported to match a sense orientation LINE L2a repeat (Figure 1). This finding represents a rare exaptation event [22, 55] but is somewhat equivocal in the context of the RepeatMasker match appearing highly degenerated and yielding a score relatively close to the Smith-Waterman cutoffs; that is 180 for the ancient MIR, L2, and MER5 sequences [41]. At this point it should be noted however that the accurate annotation of a L2 family-repeat content and the valid estimation of its original extent are issues very hard to meet since it is not unusual for these ancient, inactive elements to be degenerated beyond recognition [56, 57].

A subsequent* MYEOV* scan using PLOTREP [42] further reinforces that a segment of MYEOV-313 initial coding sequence was likely provided by a TE of the L2 family (Supplementary Data Set 3). The relatively short sequence extracted by PLOTREP, while corresponding to a minor segment of the RepeatMasker match, matches identically to the respective data extracted from the TranspoGene database and to* MYEOV* DNA segment masked by BLASTN, when a DUST-driven [44] filter for human-specific repeats is applied. Of note, RepeatMasker's results not corresponding well with the respective data extracted from PLOTREP [42], the TranspoGene database [43], and BLASTN represent not an atypical finding; this inconsistency is due to other programs using much smaller databases than RepeatMasker. RepeatMasker's mammalian libraries represent heavily manipulated and expanded versions of the respective Repbase libraries [58].

**Figure 3:**
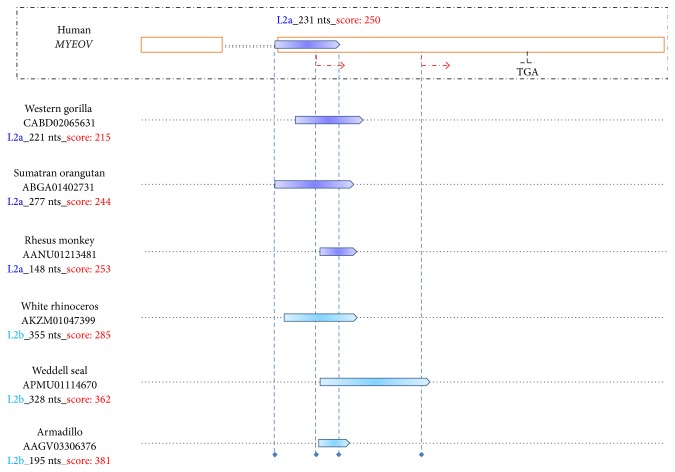
Presence of L2 family-repeat-fossils in* MYEOV* evolutionary antecedents. Upper panel: illustrated on top is* MYEOV* locus, as described in Figure 1. Shown is the location of the sense orientation L2 family-repeat followed by RepeatMasker's annotation in the context of the subfamily name appearing in consensus annotation evidence (dark blue font), the size (black font), and the Smith-Waterman score (red font) of the match. Lower panel: depicted is the presence of L2 family-repeat-relics (pentagons) in* MYEOV* syntenic region in six mammals, ranging from armadillo to western gorilla. Blue, discontinuous, vertical lines have been used to better visualize the overlap with the human repeat as well as, wherever occurring, the overlap with nonmasked human sequences. Shown in the left flank is the NCBI accession of WGS sequence contigs including the syntenic DNA sequence in each species, followed by the RepeatMasker's annotation of the degenerated repeat element included in the syntenic region. Length of white rhinoceros and armadillo repeats in the figure is not in accordance with the nucleotide content due to the presence of inserts in the syntenic region.

The above data led us to subsequently inquire into the status of the L2a repeat in* MYEOV* evolutionary antecedents. Results validated the presence of L2 family-repeat-relics in the corresponding syntenic region in, at least, seven eutherian mammals (Figure 3, Supplementary Data Set 4). Importantly, it is well known that L2s and MIRs underwent active retroposition prior to the placental mammalian radiation [22, 57]. It is also known that cases of TEs inserted independently in nearby syntenic genomic regions in species identical-by-descent represent extremely rare events with exceedingly low probability to occur [59]. In line with these data, it is highly likely that RepeatMasker not only defining divergently the repeat's size and exact boundaries in each species but also providing different subfamily names for the repeat is due to the high degree of degeneration of the repeat during its evolution in the mammalian genome. Overall, it is particularly plausible that the molecular L2 repeat-fossils, present in the nearby syntenic regions of the seven mammals (Figure 3), were derived from the same L2 transposable element that was introduced in the common eutherian ancestor.

Another line of evidence to support the above, likely the most conclusive one, would be the probabilistic reconstruction of* MYEOV* locus ancestral sequence [60]. Indeed, it has been suggested that using RepeatMasker to scan the inferred boreoeutherian ancestor sequence of a given genomic locus would provide more accurate information on the original extent of the ancient TEs included within than running the software in the corresponding human sequence [61], because the ancient repeats would appear much less degenerated in the ancestral sequence. Of note, a series of prerequisites should be met for accurately reconstructing an ancestral sequence [61]. Most importantly, problematic sampling of major lineages and outgroups seems to significantly decrease the accuracy of ancestral sequence reconstruction [61, 62]. Consequently, Myomorpha, Sciuromorpha, and Lagomorpha lacking a large segment of* MYEOV* syntenic region likely represent a severe drawback in optimally performing the sampling procedure.

### 3.3. *MYEOV* Full Splicing Potential Was Acquired Relatively Late during the Gene's Evolution in the Catarrhini Genome

**Figure 4:**
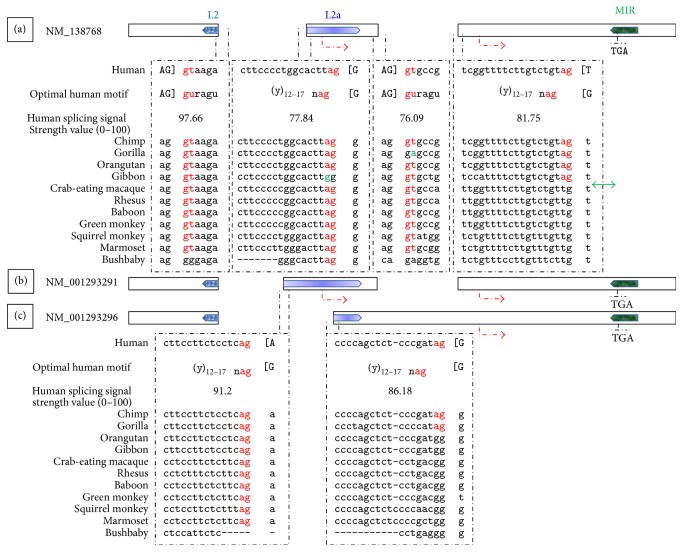
*MYEOV* splicing junctions. Illustrated are three* MYEOV* alternatively spliced variants (a–c), as described in Figure 1. Shown are the canonical 5′/3′ splice sites that delimit all the putative splicing junctions of* MYEOV*, as annotated in the Gene database from NCBI. The splice sites are juxtaposed to the optimal core splicing signal motifs; corresponding splicing signal strength values are also shown. Upper-case nucleotides, delimited by brackets, versus lower-case nucleotides correspond to exonic versus intronic content, respectively. Nucleotides exerting the strongest influence on the signal strength appear in red font. Aligned below the human splicing signals appear corresponding nucleotide sequences from* MYEOV* syntenic region in 11 primates. Shown in green font are nucleotides that deviate from the evolutionary trend, requiring further validation. The double-headed green arrow points to the precious canonical acceptor that arose in hominoids, allowing for* MYEOV* long-ORF to occur. Alignment gaps correspond to indels located between the aligned blocks in the aligning species.

As shown in Figure 4, only one* MYEOV* canonical splicing signal,* id est*, allowing for RNA processing by the standard U2 type spliceosome [63], predated the radiation of Haplorhini. Three canonical splicing signals arose in higher primates while two precious standard acceptors appeared later in time.

Importantly the canonical acceptor of* MYEOV* second splicing junction, namely, of the splicing junction allowing for* MYEOV* long-ORF to occur (Figure 1), is hominoid-specific (Figure 4).

### 3.4. Transcription of* MYEOV* Locus in Human and Nonhuman Primates

In human, transcription of* MYEOV* genomic locus is driven by a strongly active cryptic promoter sequence locating in* MYEOV* 5′ untranslated region (5′ UTR) [12], between the second and third pseudosignal of the gene (Figure 1). Should this cryptic promoter precede or follow the emergence of the translatable ORF in Catarrhini and be active in other higher primates as well remains to be clarified via subsequent studies, especially because the expressed sequence tags- (EST-) coverage of the genomic sequence in nonhuman primates is low.

Indeed, BLASTN searches with human* MYEOV* mRNA sequences against the GenBank EST database yield only one, however precious, unambiguous hit (NCBI accession: DC527428). This EST validates transcription of the genomic locus in chimpanzee.

### 3.5. MYEOV-313 Start Codon Includes Not Common Polymorphic Sites in Human

According to the single nucleotide polymorphism database from NCBI (http://www.ncbi.nlm.nih.gov/snp/), located in human* MYEOV* gene are 17 common polymorphic sites. None locates in MYEOV-313 start codon.

In the same context, out of the 7 common polymorphic sites located in* MYEOV* long-ORF, all representing single nucleotide substitutions, none induces a nonsense codon. Out of the 6 common polymorphisms included in either* MYEOV* 5′ UTR or* MYEOV* intragenic regions, none locates within core splicing signals.

In this context, it is highly plausible that all individuals could be able to encode for the 313-aa peptide.

### 3.6. *MYEOV* Peptide(s) May Be Involved* Per Se* in Promoting the Malignant State

In line with data extracted from COSMIC [48], only 60* MYEOV* confirmed somatic mutations were identified in 72 out of 23780 unique samples tested.

The above could signify that the peptide(s) produced by* MYEOV* may be involved* per se*, rather than mutated, in promoting the malignant state.

## 4. Conclusions

(i)* MYEOV* is a Primate Orphan Gene that acquired, via ORF expansion, a human-protein-specific coding potential. Thus it represents the first cancer-related gene identified to present significantly differing protein-coding potential between human and chimpanzee.

(ii) Should* MYEOV* role in promoting cancer cell proliferation and metastasis be attributed to MYEOV-313 is a tantalizing question that warrants further investigation, especially in the context that all individuals could be able to encode for this peptide and also because MYEOV-313 may be related* per se* in promoting tumour propagation and thus could be specifically targeted for. After all, targeting a human-specific peptide would theoretically cause less adverse effects than targeting components of evolutionary conserved signaling cascades.

(iii) It was adaptive evolution coupled with TE exaptation that allowed for the expansion of* MYEOV* pORF. It is tempting to speculate that it could have all started with the relatively strong core splicing signals provided by the LINE L2a repeat (Figure 4), creating an evolutionary “hotspot” within* MYEOV* sequence that was subsequently positively selected for throughout the course of Catarrhini evolution [64].

## Supplementary Material

Supplementary Material includes one Figure and four Data Sets. Depicted in Supplementary Figure 1 is that the DNA segment where *MYEOV* locates emerged in Eutherians. Supplementary Data Set 1 includes sequence data from selected segments in *MYEOV* syntenic region in numerous Vertebrates, extracted from the "Multiz Alignments of 100 Vertebrates" track of the UCSC Genome Browser Database (http://genome.ucsc.edu/), reinforcing the results reported in the text. Supplementary Data Set 2 includes the results of *MYEOV* genomic sequence scan by Repeat Masker; run in both default and sensitive mode. Supplementary Data Set 3 contains the short *MYEOV* coding sequence extracted identically from PLOTREP, TranspoGene and BLASTN as corresponding to a L2 repeat segment. Supplementary Data Set 4 includes RepeatMasker results verifying the presence of L2 family repeat-relics in *MYEOV* syntenic region in six eutherian mammals

## Data Access

The sequence data from this study are archived at the Gene database from NCBI (http://www.ncbi.nlm.nih.gov/gene/), the Ensembl Genome Browser Database (http://www.ensembl.org/index.html), and the UCSC Genome Browser Database (http://genome.ucsc.edu/).
