# Supplementary material for: Adaptive Evolution Coupled with Retrotransposon Exaptation Allowed for the Generation of a Human-Protein-Specific Coding Gene That Promotes Cancer Cell Proliferation and Metastasis in Both Haematological Malignancies and Solid Tumours: The Extraordinary Case of MYEOV Gene
Source: Scientifica (Cairo). 2015 Oct 19;2015:984706. doi: 10.1155/2015/984706 (PMC4629056; doi:10.1155/2015/984706)
Supplement: Supplementary file 1 — Supplementary Material includes one Figure and four Data Sets. Depicted in Supplementary Figure 1 is that the DNA segment where MYEOV locates emerged in Eutherians. Supplementary Data Set 1 includes sequence data from selected segments in MYEOV syntenic region in numerous Vertebrates, extracted from the "Multiz Alignments of 100 Vertebrates" track of the UCSC Genome Browser Database (http://genome.ucsc.edu/), reinforcing the results reported in the text. Supplementary Data Set 2 includes the results of MYEOV genomic sequence scan by Repeat Masker; run in both default and sensitive mode. Supplementary Data Set 3 contains the short MYEOV coding sequence extracted identically from PLOTREP, TranspoGene and BLASTN as corresponding to a L2 repeat segment. Supplementary Data Set 4 includes RepeatMasker results verifying the presence of L2 family repeat-relics in MYEOV syntenic region in six eutherian mammals [file 984706.f1.docx]

**Supplementary Material**

**This file includes:**

**Supplementary Figure 1**

**Supplementary Data Sets 1-4**


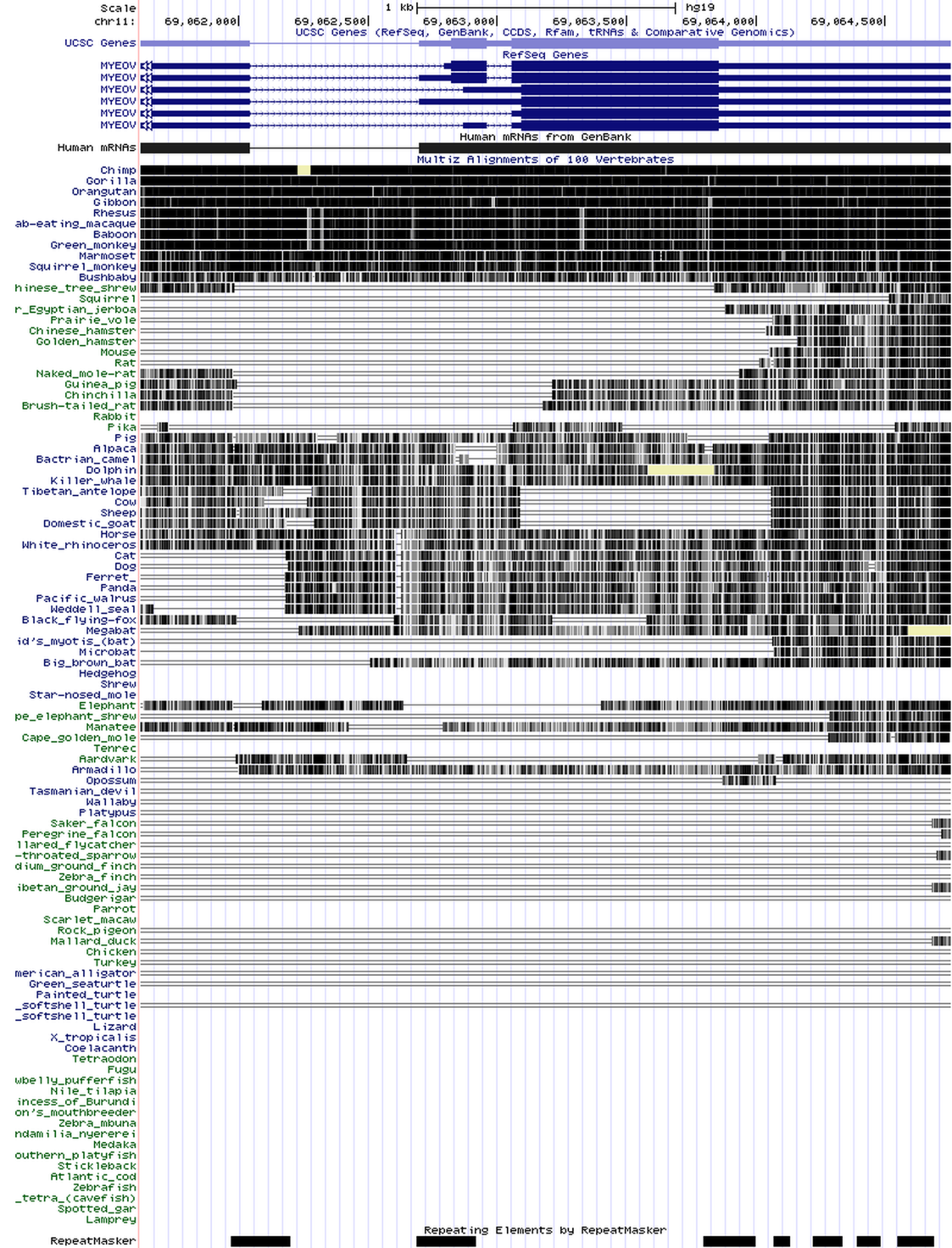


**Supplementary Figure 1**

Visualized are syntenic alignments among numerous Vertebrates supporting that the DNA segment where *MYEOV* locates emerged in eutherians. The figure was extracted from the “RefSeq Genes/ Human mRNAs / Multiz Alignments of 100 Vertebrates/ RepeatMasker” tracks of the UCSC Genome Browser Database (<http://genome.ucsc.edu/>) and is shown unmodified; conservation track set was configured to include all the Vertebrate species available in the database.

**Supplementary Data Sets 1-4**

**Supplementary Data Set 1**

Sequence data extracted from the “Multiz Alignments of 100 Vertebrates” track of the UCSC Genome Browser Database (<http://genome.ucsc.edu/>), showing that:

(I) The *ATG* trinucleotide corresponding to MYEOV-313 start codon is exclusively present in human and white-tufted-ear marmoset.

(II) MYEOV-255 start codon arose in Catarrhini.

(III) The 2 bp insertion (*GA*) in Sumatran orangutan *MYEOV* ortholog, shifting very early the respective pORF, is species-specific. Bushbaby suffered also a 2 bp (*GT*) insertion likely due to a parallel mutation.

(IV) The Cercopithecoidea-specific 14 bp deletion resulted in the in-frame translocation of a, fixed exclusively in Cercopithecoidea, *TGA* trinucleotide.

**Supplementary Data Set 2**

Results of *MYEOV* genomic sequence scan by Repeat Masker; run in both default (A) and sensitive (B) mode using a matrix optimal for *MYEOV* GC level. The start and stop codons of MYEOV-313 protein are highlighted in red. Core splicing signals of the genomic sequence appear underlined in red font. Highlighted in yellow are the size and the Smith-Waterman score of the equivocal fragment of the L2a repeat.

**Supplementary Data Set 3**

The short *MYEOV* coding sequence extracted - identically- from PLOTREP (Program: Censor; Library type: Repbase Update; Database: Human repetitive elements [v17.09]; Options: default settings), TranspoGene and BLASTN as corresponding to a L2 repeat segment.

**Supplementary Data Set 4**

RepeatMasker results verifying the presence of L2 family repeat-relics in nearby syntenic regions in western gorilla (I), sumatran orangutan (II), rhesus monkey (III), white rhinoceros (IV), weddell seal (V) and armadillo (VI). Reported in author’s notes is the NCBI accession of each species WGS sequence contig that includes within the DNA segment aligning to human *MYEOV.*

**Supplementary Data Set 1**

**(I)**

**Alignment block 34 of 70 in window, 69062793 - 69062831, 39 bps**

[B](http://genome.ucsc.edu/cgi-bin/hgTracks?db=hg19&ct=&position=chr11%3A69062793-69062831) [D](http://genome.ucsc.edu/cgi-bin/hgc?o=69062792&g=getDna&i=chr11&c=chr11&l=69062792&r=69062831&db=hg19) Human ggac----------agc-----------------gtctggctccttccctcgg--ctc**A**--**T**--------

[B](http://genome.ucsc.edu/cgi-bin/hgTracks?db=panTro4&ct=&position=chr11%3A67008256-67008294) [D](http://genome.ucsc.edu/cgi-bin/hgc?o=67008255&g=getDna&i=chr11&c=chr11&l=67008255&r=67008294&db=panTro4) Chimp ggac----------agc-----------------gtctggctccttccctcgg--ctcG--T--------

[B](http://genome.ucsc.edu/cgi-bin/hgTracks?db=gorGor3&ct=&position=chr11%3A66278210-66278248) [D](http://genome.ucsc.edu/cgi-bin/hgc?o=66278209&g=getDna&i=chr11&c=chr11&l=66278209&r=66278248&db=gorGor3) Gorilla ggac----------agc-----------------gtctggctccttccctcag--ctcG--T--------

[B](http://genome.ucsc.edu/cgi-bin/hgTracks?db=ponAbe2&ct=&position=chr11%3A6739707-6739745) [D](http://genome.ucsc.edu/cgi-bin/hgc?o=6739706&g=getDna&i=chr11&c=chr11&l=6739706&r=6739745&db=ponAbe2&hgSeq.revComp=on) Orangutan ggac----------agc-----------------gtctagctccttccctcag--ctcG--T--------

[B](http://genome.ucsc.edu/cgi-bin/hgTracks?db=nomLeu3&ct=&position=chr4%3A89527561-89527599) [D](http://genome.ucsc.edu/cgi-bin/hgc?o=89527560&g=getDna&i=chr4&c=chr4&l=89527560&r=89527599&db=nomLeu3) Gibbon ggac----------agt-----------------gtctggctccttcccttgg--ctcG--T--------

[B](http://genome.ucsc.edu/cgi-bin/hgTracks?db=rheMac3&ct=&position=chr14%3A5312453-5312491) [D](http://genome.ucsc.edu/cgi-bin/hgc?o=5312452&g=getDna&i=chr14&c=chr14&l=5312452&r=5312491&db=rheMac3&hgSeq.revComp=on) Rhesus ggac----------agc-----------------atctggctccttccctcgg--ctgG--T--------

Crab-eating macaque ggac----------agc-----------------atctggctccttccctcgg--ctgG--T--------

[B](http://genome.ucsc.edu/cgi-bin/hgTracks?db=papHam1&ct=&position=scaffold26046%3A3649-3687) [D](http://genome.ucsc.edu/cgi-bin/hgc?o=3648&g=getDna&i=scaffold26046&c=scaffold26046&l=3648&r=3687&db=papHam1) Baboon ggac----------agc-----------------atctggctccttccctcgg--ctgG--T--------

Green monkey ggac----------agc-----------------atctggctccttccctcgg--ctcG--T--------

[B](http://genome.ucsc.edu/cgi-bin/hgTracks?db=calJac3&ct=&position=chr11_GL285516_random%3A3651-3689) [D](http://genome.ucsc.edu/cgi-bin/hgc?o=3650&g=getDna&i=chr11_GL285516_random&c=chr11_GL285516_random&l=3650&r=3689&db=calJac3) Marmoset ggac----------agc-----------------gtctggctccttccctcag--ctc**A**--**T**--------

[B](http://genome.ucsc.edu/cgi-bin/hgTracks?db=saiBol1&ct=&position=JH378247%3A2100934-2100972) [D](http://genome.ucsc.edu/cgi-bin/hgc?o=2100933&g=getDna&i=JH378247&c=JH378247&l=2100933&r=2100972&db=saiBol1&hgSeq.revComp=on) Squirrel monkey ggac----------agt-----------------gtctggctccttcccttgg--ctcG--T--------

[B](http://genome.ucsc.edu/cgi-bin/hgTracks?db=otoGar3&ct=&position=GL873653%3A1413499-1413539) [D](http://genome.ucsc.edu/cgi-bin/hgc?o=1413498&g=getDna&i=GL873653&c=GL873653&l=1413498&r=1413539&db=otoGar3&hgSeq.revComp=on) Bushbaby ggac----------aac-----------------atctggctcctttcctgggcccccG--T--------

[B](http://genome.ucsc.edu/cgi-bin/hgTracks?db=susScr3&ct=&position=chr2%3A2576915-2576954) [D](http://genome.ucsc.edu/cgi-bin/hgc?o=2576914&g=getDna&i=chr2&c=chr2&l=2576914&r=2576954&db=susScr3&hgSeq.revComp=on) Pig ggtc----------cgt-----------------gtctg-----ctctgagca--ccc---GGAGGGCT-

[B](http://genome.ucsc.edu/cgi-bin/hgTracks?db=vicPac2&ct=&position=KB632549%3A2270849-2270887) [D](http://genome.ucsc.edu/cgi-bin/hgc?o=2270848&g=getDna&i=KB632549&c=KB632549&l=2270848&r=2270887&db=vicPac2) Alpaca gaac----------ggt-----------------gtcca-----ttccgcatg--tggA--CGAGTGC--

Bactrian camel gaac----------ggt-----------------gtcca-----ttccgcatg--cggA--CGAG-----

[B](http://genome.ucsc.edu/cgi-bin/hgTracks?db=turTru2&ct=&position=JH472550%3A248372-248419) [D](http://genome.ucsc.edu/cgi-bin/hgc?o=248371&g=getDna&i=JH472550&c=JH472550&l=248371&r=248419&db=turTru2) Dolphin ggacaatgtcc---ggt-----------------gtcta-----ctccacacg--cccT--GGAGGGCC-

Killer whale ggacaatgtcc---ggt-----------------gtcta-----ctccacacg--cccT--GGAGGGCC-

Tibetan antelope gaacaatggccacggat-----------------gtccg-----ctccacgtg--cccT--GGAGGGCT-

[B](http://genome.ucsc.edu/cgi-bin/hgTracks?db=bosTau7&ct=&position=chrUn_JH121853%3A27306-27356) [D](http://genome.ucsc.edu/cgi-bin/hgc?o=27305&g=getDna&i=chrUn_JH121853&c=chrUn_JH121853&l=27305&r=27356&db=bosTau7) Cow gaacaatggccacggat-----------------gtccg-----ctccacgtg--cccT--GGAGGGCT-

[B](http://genome.ucsc.edu/cgi-bin/hgTracks?db=oviAri3&ct=&position=chr21%3A45884789-45884839) [D](http://genome.ucsc.edu/cgi-bin/hgc?o=45884788&g=getDna&i=chr21&c=chr21&l=45884788&r=45884839&db=oviAri3) Sheep gaacaatggccacggat-----------------gtccg-----ctccacgtg--cccT--GGAGGGCT-

Domestic goat gaacaatggccacggat-----------------gtccg-----ctccatgtg--cccT--GGAGGGCT-

[B](http://genome.ucsc.edu/cgi-bin/hgTracks?db=equCab2&ct=&position=chr12%3A28435441-28435482) [D](http://genome.ucsc.edu/cgi-bin/hgc?o=28435440&g=getDna&i=chr12&c=chr12&l=28435440&r=28435482&db=equCab2) Horse gggt----------gat-----------------gtccc-----ctccacgag--accC--TGGGGGGCT

[B](http://genome.ucsc.edu/cgi-bin/hgTracks?db=cerSim1&ct=&position=JH767824%3A4854141-4854182) [D](http://genome.ucsc.edu/cgi-bin/hgc?o=4854140&g=getDna&i=JH767824&c=JH767824&l=4854140&r=4854182&db=cerSim1&hgSeq.revComp=on) White rhinoceros ggat----------gat-----------------gtctg-----ctccacgag--gccC--TGGAGGTCC

[B](http://genome.ucsc.edu/cgi-bin/hgTracks?db=felCat5&ct=&position=chrD1%3A112914186-112914233) [D](http://genome.ucsc.edu/cgi-bin/hgc?o=112914185&g=getDna&i=chrD1&c=chrD1&l=112914185&r=112914233&db=felCat5) Cat ggac----------agt----------------tgtcca-----ccccacgag--gccC--TGGAGGTCT

[B](http://genome.ucsc.edu/cgi-bin/hgTracks?db=canFam3&ct=&position=chr18%3A48782563-48782595) [D](http://genome.ucsc.edu/cgi-bin/hgc?o=48782562&g=getDna&i=chr18&c=chr18&l=48782562&r=48782595&db=canFam3&hgSeq.revComp=on) Dog ggat----------ggt----------------tgtcca-----ctccatgag--gc-C--T--------

[B](http://genome.ucsc.edu/cgi-bin/hgTracks?db=musFur1&ct=&position=GL896969%3A6099845-6099879) [D](http://genome.ucsc.edu/cgi-bin/hgc?o=6099844&g=getDna&i=GL896969&c=GL896969&l=6099844&r=6099879&db=musFur1) Ferret ggat----------agt----------------tgtcca-----ctccatgag--gccC--T--------

[B](http://genome.ucsc.edu/cgi-bin/hgTracks?db=ailMel1&ct=&position=GL193710.1%3A134945-134979) [D](http://genome.ucsc.edu/cgi-bin/hgc?o=134944&g=getDna&i=GL193710.1&c=GL193710.1&l=134944&r=134979&db=ailMel1) Panda ggac----------agt----------------tctcca-----tgccacgag--gccC--T--------

Pacific walrus gggc----------agt----------------cgtcca-----ctccatgag--gcgC--T--------

Weddell seal ggac----------agt----------------cgtcta-----ctccatgag--gccC--T--------

Black flying-fox ggcc----------cgc-------------ctgggtctg-----tcc-----------------------

[B](http://genome.ucsc.edu/cgi-bin/hgTracks?db=pteVam1&ct=&position=scaffold_27084%3A3559-3578) [D](http://genome.ucsc.edu/cgi-bin/hgc?o=3558&g=getDna&i=scaffold_27084&c=scaffold_27084&l=3558&r=3578&db=pteVam1&hgSeq.revComp=on) Megabat ggcc----------cgc-------------ctgggtctg-----tcc-----------------------

Big brown bat gcac----------catgagatcccaggggcttggcctc-----cct-----------------------

[B](http://genome.ucsc.edu/cgi-bin/hgTracks?db=triMan1&ct=&position=JH594719%3A2461817-2461853) [D](http://genome.ucsc.edu/cgi-bin/hgc?o=2461816&g=getDna&i=JH594719&c=JH594719&l=2461816&r=2461853&db=triMan1&hgSeq.revComp=on) Manatee ggcc----------aaa-----------------gttagact--gtcacacag--cccA--A--------

[B](http://genome.ucsc.edu/cgi-bin/hgTracks?db=dasNov3&ct=&position=JH583290%3A23561-23636) [D](http://genome.ucsc.edu/cgi-bin/hgc?o=23560&g=getDna&i=JH583290&c=JH583290&l=23560&r=23636&db=dasNov3&hgSeq.revComp=on) Armadillo ggac----------agc-----------------atc-ggct--ctcctgcag--cccACGA--------

Pika ======================================================================

Cape elephant shrew ======================================================================

[B](http://genome.ucsc.edu/cgi-bin/hgTracks?db=mm10&ct=&position=chr7%3A145112604-145113522) [D](http://genome.ucsc.edu/cgi-bin/hgc?o=145112603&g=getDna&i=chr7&c=chr7&l=145112603&r=145113522&db=mm10&hgSeq.revComp=on) Mouse ======================================================================

Prairie vole ======================================================================

[B](http://genome.ucsc.edu/cgi-bin/hgTracks?db=rn5&ct=&position=chr1%3A225141922-225142822) [D](http://genome.ucsc.edu/cgi-bin/hgc?o=225141921&g=getDna&i=chr1&c=chr1&l=225141921&r=225142822&db=rn5&hgSeq.revComp=on) Rat ======================================================================

Cape golden mole ======================================================================

[B](http://genome.ucsc.edu/cgi-bin/hgTracks?db=criGri1&ct=&position=KE378474%3A1855202-1856134) [D](http://genome.ucsc.edu/cgi-bin/hgc?o=1855201&g=getDna&i=KE378474&c=KE378474&l=1855201&r=1856134&db=criGri1) Chinese hamster ======================================================================

Golden hamster ======================================================================

Aardvark ======================================================================

Lesser Egyptian jerboa ======================================================================

Brush-tailed rat ======================================================================

Chinchilla ======================================================================

[B](http://genome.ucsc.edu/cgi-bin/hgTracks?db=cavPor3&ct=&position=scaffold_42%3A9185303-9185540) [D](http://genome.ucsc.edu/cgi-bin/hgc?o=9185302&g=getDna&i=scaffold_42&c=scaffold_42&l=9185302&r=9185540&db=cavPor3) Guinea pig ======================================================================

White-throated sparrow ======================================================================

[B](http://genome.ucsc.edu/cgi-bin/hgTracks?db=geoFor1&ct=&position=JH739992%3A1483124-1483123) [D](http://genome.ucsc.edu/cgi-bin/hgc?o=1483123&g=getDna&i=JH739992&c=JH739992&l=1483123&r=1483123&db=geoFor1) Medium ground finch ======================================================================

Peregrine falcon ======================================================================

Saker falcon ======================================================================

Collared flycatcher ======================================================================

Tibetan ground jay ======================================================================

Mallard duck ======================================================================

[B](http://genome.ucsc.edu/cgi-bin/hgTracks?db=melUnd1&ct=&position=JH556606%3A2045680-2045679) [D](http://genome.ucsc.edu/cgi-bin/hgc?o=2045679&g=getDna&i=JH556606&c=JH556606&l=2045679&r=2045679&db=melUnd1) Budgerigar ======================================================================

Rock pigeon ======================================================================

[B](http://genome.ucsc.edu/cgi-bin/hgTracks?db=myoLuc2&ct=&position=GL429985%3A167075-167074) [D](http://genome.ucsc.edu/cgi-bin/hgc?o=167074&g=getDna&i=GL429985&c=GL429985&l=167074&r=167074&db=myoLuc2) Microbat ----------------------------------------------------------------------

David's myotis (bat) ======================================================================

[B](http://genome.ucsc.edu/cgi-bin/hgTracks?db=loxAfr3&ct=&position=scaffold_71%3A509092-509091) [D](http://genome.ucsc.edu/cgi-bin/hgc?o=509091&g=getDna&i=scaffold_71&c=scaffold_71&l=509091&r=509091&db=loxAfr3&hgSeq.revComp=on) Elephant ----------------------------------------------------------------------

[B](http://genome.ucsc.edu/cgi-bin/hgTracks?db=speTri2&ct=&position=JH393409%3A1184098-1185245) [D](http://genome.ucsc.edu/cgi-bin/hgc?o=1184097&g=getDna&i=JH393409&c=JH393409&l=1184097&r=1185245&db=speTri2&hgSeq.revComp=on) Squirrel ======================================================================

Chinese tree shrew ======================================================================

[B](http://genome.ucsc.edu/cgi-bin/hgTracks?db=hetGla2&ct=&position=JH602080%3A17761395-17762045) [D](http://genome.ucsc.edu/cgi-bin/hgc?o=17761394&g=getDna&i=JH602080&c=JH602080&l=17761394&r=17762045&db=hetGla2&hgSeq.revComp=on) Naked mole-rat ======================================================================

[B](http://genome.ucsc.edu/cgi-bin/hgTracks?db=ornAna1&ct=&position=chr3%3A7531224-7590302) [D](http://genome.ucsc.edu/cgi-bin/hgc?o=7531223&g=getDna&i=chr3&c=chr3&l=7531223&r=7590302&db=ornAna1) Platypus ======================================================================

[B](http://genome.ucsc.edu/cgi-bin/hgTracks?db=macEug2&ct=&position=GL105664%3A20690-59466) [D](http://genome.ucsc.edu/cgi-bin/hgc?o=20689&g=getDna&i=GL105664&c=GL105664&l=20689&r=59466&db=macEug2) Wallaby ======================================================================

Soft-shell Turtle ======================================================================

Green seaturtle ======================================================================

[B](http://genome.ucsc.edu/cgi-bin/hgTracks?db=melGal1&ct=&position=chr5%3A17376478-17407513) [D](http://genome.ucsc.edu/cgi-bin/hgc?o=17376477&g=getDna&i=chr5&c=chr5&l=17376477&r=17407513&db=melGal1) Turkey ======================================================================

[B](http://genome.ucsc.edu/cgi-bin/hgTracks?db=taeGut2&ct=&position=chr5%3A4972475-5001515) [D](http://genome.ucsc.edu/cgi-bin/hgc?o=4972474&g=getDna&i=chr5&c=chr5&l=4972474&r=5001515&db=taeGut2) Zebra finch ======================================================================

[B](http://genome.ucsc.edu/cgi-bin/hgTracks?db=sarHar1&ct=&position=chr6_GL864908_random%3A330350-372620) [D](http://genome.ucsc.edu/cgi-bin/hgc?o=330349&g=getDna&i=chr6_GL864908_random&c=chr6_GL864908_random&l=330349&r=372620&db=sarHar1) Tasmanian devil ======================================================================

[B](http://genome.ucsc.edu/cgi-bin/hgTracks?db=galGal4&ct=&position=chr5%3A16527834-16527833) [D](http://genome.ucsc.edu/cgi-bin/hgc?o=16527833&g=getDna&i=chr5&c=chr5&l=16527833&r=16527833&db=galGal4&hgSeq.revComp=on) Chicken ======================================================================

[B](http://genome.ucsc.edu/cgi-bin/hgTracks?db=allMis1&ct=&position=JH737694%3A164551-223805) [D](http://genome.ucsc.edu/cgi-bin/hgc?o=164550&g=getDna&i=JH737694&c=JH737694&l=164550&r=223805&db=allMis1) American alligator ======================================================================

[B](http://genome.ucsc.edu/cgi-bin/hgTracks?db=monDom5&ct=&position=chr5%3A304021248-304050406) [D](http://genome.ucsc.edu/cgi-bin/hgc?o=304021247&g=getDna&i=chr5&c=chr5&l=304021247&r=304050406&db=monDom5) Opossum ======================================================================

Human -----**G**GCCCTC--------------------------------------A

Chimp -----GGCCCTC--------------------------------------A

Gorilla -----GGCCCTC--------------------------------------A

Orangutan -----GGCCCTT--------------------------------------A

Gibbon -----GGCCCTC--------------------------------------A

Rhesus -----GGCCCTC--------------------------------------A

Crab-eating macaque -----GGCCCTC--------------------------------------A

Baboon -----GGCCCTC--------------------------------------A

Green monkey -----GGCCCTC--------------------------------------A

Marmoset -----**G**GCCCTC--------------------------------------A

Squirrel monkey -----GGCCCTC--------------------------------------A

Bushbaby -----GGCCCCC--------------------------------------T

Pig -----GGCCTGT--------------------------------------A

Alpaca ------GCCAGC--------------------------------------T

Bactrian camel ---------------------------------------------------

Dolphin -----GGCCCGC--------------------------------------C

Killer whale -----GGCCCGC--------------------------------------C

Tibetan antelope -----GGCCCGC--------------------------------------C

Cow -----GGCCCGC--------------------------------------G

Sheep -----GGCCCGC--------------------------------------C

Domestic goat -----GGCCCGC--------------------------------------C

Horse -----GGCCTTC--------------------------------------C

White rhinoceros -----AGCCTGC--------------------------------------C

Cat GTCCCGGCCTGC--------------------------------------C

Dog -----GGCCT-C--------------------------------------C

Ferret -----GGCCTGC--------------------------------------C

Panda -----GGCCTGT--------------------------------------C

Pacific walrus -----GGCCTGC--------------------------------------C

Weddell seal -----GGCCTGC--------------------------------------C

Black flying-fox -----G---------------------------------------------

Megabat -----G---------------------------------------------

Big brown bat -----GGGCTG----------------------------------------

Manatee -----GGCCCCC--------------------------------------A

Armadillo -----GGCCCCTGGAGGTCACGCCGTCCCCAGCCACACCCTCCCACCCTGA

Pika ===================================================

Cape elephant shrew ===================================================

Mouse ===================================================

Prairie vole ===================================================

Rat ===================================================

Cape golden mole ===================================================

Chinese hamster ===================================================

Golden hamster ===================================================

Aardvark ===================================================

Lesser Egyptian jerboa ===================================================

Brush-tailed rat ===================================================

Chinchilla ===================================================

Guinea pig ===================================================

White-throated sparrow ===================================================

Medium ground finch ===================================================

Peregrine falcon ===================================================

Saker falcon ===================================================

Collared flycatcher ===================================================

Tibetan ground jay ===================================================

Mallard duck ===================================================

Budgerigar ===================================================

Rock pigeon ===================================================

Microbat ---------------------------------------------------

David's myotis (bat) ===================================================

Elephant ---------------------------------------------------

Squirrel ===================================================

Chinese tree shrew ===================================================

Naked mole-rat ===================================================

Platypus ===================================================

Wallaby ===================================================

Soft-shell Turtle ===================================================

Green seaturtle ===================================================

Turkey ===================================================

Zebra finch ===================================================

Tasmanian devil ===================================================

Chicken ===================================================

American alligator ===================================================

Opossum ===================================================

**(II)**

**Alignment block 41 of 70 in window, 69063081 - 69063178, 98 bps**

[B](http://genome.ucsc.edu/cgi-bin/hgTracks?db=hg19&ct=&position=chr11%3A69063081-69063178) [D](http://genome.ucsc.edu/cgi-bin/hgc?o=69063080&g=getDna&i=chr11&c=chr11&l=69063080&r=69063178&db=hg19) Human ACTCGTTGCTCATGTTCACC--CGGCAGGCTG---GACACTTCGT--GGAGGGCTCCAAAGCCGGCAGAT

[B](http://genome.ucsc.edu/cgi-bin/hgTracks?db=panTro4&ct=&position=chr11%3A67008544-67008641) [D](http://genome.ucsc.edu/cgi-bin/hgc?o=67008543&g=getDna&i=chr11&c=chr11&l=67008543&r=67008641&db=panTro4) Chimp ACTCGTTGCTCATGTTCACC--CGGCAGGCTG---GACGCTTCAT--GGAGGGCTCCAAAGCTGGCAGAT

[B](http://genome.ucsc.edu/cgi-bin/hgTracks?db=gorGor3&ct=&position=chr11%3A66278498-66278595) [D](http://genome.ucsc.edu/cgi-bin/hgc?o=66278497&g=getDna&i=chr11&c=chr11&l=66278497&r=66278595&db=gorGor3) Gorilla ACTTGTTGCTCATGTTCACC--TGGCAGGCTG---GACGCTTCGT--GGAGGGCTCCAAAGCTGGCAGAT

[B](http://genome.ucsc.edu/cgi-bin/hgTracks?db=ponAbe2&ct=&position=chr11%3A6739360-6739457) [D](http://genome.ucsc.edu/cgi-bin/hgc?o=6739359&g=getDna&i=chr11&c=chr11&l=6739359&r=6739457&db=ponAbe2&hgSeq.revComp=on) Orangutan ACTGGTCACTCATGTTCATC--CGGCAGGCTG---GATGCTTCGT--GGAGGGCTCCAAAGCTGGCAGAC

[B](http://genome.ucsc.edu/cgi-bin/hgTracks?db=nomLeu3&ct=&position=chr4%3A89527835-89527932) [D](http://genome.ucsc.edu/cgi-bin/hgc?o=89527834&g=getDna&i=chr4&c=chr4&l=89527834&r=89527932&db=nomLeu3) Gibbon ACTGGTTGCTCATGTTCATC--CGGCAGGCTG---GACGCTTCGT--GGAGGGCTCCAAAGCTGGCAGAT

[B](http://genome.ucsc.edu/cgi-bin/hgTracks?db=rheMac3&ct=&position=chr14%3A5312106-5312203) [D](http://genome.ucsc.edu/cgi-bin/hgc?o=5312105&g=getDna&i=chr14&c=chr14&l=5312105&r=5312203&db=rheMac3&hgSeq.revComp=on) Rhesus ACTGGTCACTCATGTTCATC--CGGCAGACCA---GACACTTCGT--GGAGGGCTCCAAAGCTGGCACAT

Crab-eating macaque ACTGGTCACTCATGTTCATC--CGGCAGACCA---GACACTTCGT--GGAGGGCTCCAAAGCTGGCACAT

[B](http://genome.ucsc.edu/cgi-bin/hgTracks?db=papHam1&ct=&position=scaffold26046%3A3937-4034) [D](http://genome.ucsc.edu/cgi-bin/hgc?o=3936&g=getDna&i=scaffold26046&c=scaffold26046&l=3936&r=4034&db=papHam1) Baboon ACTGGTCACTCATGTTCATC--CAGCAGACCG---GACACTTCGT--GGAGGGCTCCAAAGCTGGCACAT

Green monkey ACTGGATACTCATGTTCATC--CGGCAGACCG---GACACTTTGT--GGAGGGCTCCAAAGCTGGCACAT

[B](http://genome.ucsc.edu/cgi-bin/hgTracks?db=calJac3&ct=&position=chr11_GL285516_random%3A3937-4032) [D](http://genome.ucsc.edu/cgi-bin/hgc?o=3936&g=getDna&i=chr11_GL285516_random&c=chr11_GL285516_random&l=3936&r=4032&db=calJac3) Marmoset ACTGGTCAC-CGTTTTCACC--TGGTAGGCTG---GACGCTTCGT--GGAGGGCTCCAAAGCTGGCCGAT

[B](http://genome.ucsc.edu/cgi-bin/hgTracks?db=saiBol1&ct=&position=JH378247%3A2100573-2100668) [D](http://genome.ucsc.edu/cgi-bin/hgc?o=2100572&g=getDna&i=JH378247&c=JH378247&l=2100572&r=2100668&db=saiBol1&hgSeq.revComp=on) Squirrel monkey ACCGGTCAC-TGTGTTCACC--CGGTAGGCTG---GATGCTTCAT--GGAGGGCTCCAAAGCGGGCCGAT

[B](http://genome.ucsc.edu/cgi-bin/hgTracks?db=otoGar3&ct=&position=GL873653%3A1413117-1413212) [D](http://genome.ucsc.edu/cgi-bin/hgc?o=1413116&g=getDna&i=GL873653&c=GL873653&l=1413116&r=1413212&db=otoGar3&hgSeq.revComp=on) Bushbaby CCCGGTCACTCTTGCCAACC--CTGCAAGTGG---GACA--TCTT--GGAAGGCTCCCAGGCTGG--GCT

Pika GCTTGACCCATCTACCGGCA--CAGCAGCCTG---GACACGCCGT--GGAGTACTCCA---CAGGCA---

[B](http://genome.ucsc.edu/cgi-bin/hgTracks?db=susScr3&ct=&position=chr2%3A2576559-2576642) [D](http://genome.ucsc.edu/cgi-bin/hgc?o=2576558&g=getDna&i=chr2&c=chr2&l=2576558&r=2576642&db=susScr3&hgSeq.revComp=on) Pig ---------GCTTGTCCAGT--GGGCGG-------GATGTTCCGTGGGGGGGGACCCAAAGCTGG--GCC

[B](http://genome.ucsc.edu/cgi-bin/hgTracks?db=vicPac2&ct=&position=KB632549%3A2270949-2271040) [D](http://genome.ucsc.edu/cgi-bin/hgc?o=2270948&g=getDna&i=KB632549&c=KB632549&l=2270948&r=2271040&db=vicPac2) Alpaca ACTGGCTCTTCTTTCCCACT--TGACAG-------GATGCTCCGT--GGGGGGATCCAAAACTGG--GCC

Bactrian camel GCTGGCTCTTCTTTCCCGCT--TGACAG-------GATGCTCCGT--GGGGGGATCCAAAACTGG--GCC

[B](http://genome.ucsc.edu/cgi-bin/hgTracks?db=turTru2&ct=&position=JH472550%3A248690-248750) [D](http://genome.ucsc.edu/cgi-bin/hgc?o=248689&g=getDna&i=JH472550&c=JH472550&l=248689&r=248750&db=turTru2) Dolphin -----------------------GGCAG-------GATGCTCCGT--GGAGGGATCCAAAGCTGG--GCC

Killer whale -----------------------GGCAG-------GATGCTCCGT--GGAGGGATCCAAAGCTGG--GCC

[B](http://genome.ucsc.edu/cgi-bin/hgTracks?db=equCab2&ct=&position=chr12%3A28435809-28435912) [D](http://genome.ucsc.edu/cgi-bin/hgc?o=28435808&g=getDna&i=chr12&c=chr12&l=28435808&r=28435912&db=equCab2) Horse ACTAGCTCTTCTTGTCCCCT--CGGCGGGCTGGTGGATGCCCTGC--GGAGGGATCCAAAGCTGG--GCC

[B](http://genome.ucsc.edu/cgi-bin/hgTracks?db=cerSim1&ct=&position=JH767824%3A4853711-4853800) [D](http://genome.ucsc.edu/cgi-bin/hgc?o=4853710&g=getDna&i=JH767824&c=JH767824&l=4853710&r=4853800&db=cerSim1&hgSeq.revComp=on) White rhinoceros AATGACAATGAAAG------------GGGCTAGTGGACGCCCCAT--GCAGGGATCCAAAGCTGG--ACC

[B](http://genome.ucsc.edu/cgi-bin/hgTracks?db=felCat5&ct=&position=chrD1%3A112914511-112914608) [D](http://genome.ucsc.edu/cgi-bin/hgc?o=112914510&g=getDna&i=chrD1&c=chrD1&l=112914510&r=112914608&db=felCat5) Cat TCTGGCTCCCCTTGTCCACT--GGGCGGGA-GGCACACGTTCCAC--GGAGAGATCCAAA-CTGG--GGC

[B](http://genome.ucsc.edu/cgi-bin/hgTracks?db=canFam3&ct=&position=chr18%3A48782166-48782266) [D](http://genome.ucsc.edu/cgi-bin/hgc?o=48782165&g=getDna&i=chr18&c=chr18&l=48782165&r=48782266&db=canFam3&hgSeq.revComp=on) Dog ATTGGCTCCTCTTGTCCCCGCAGGCCGGGCTGGTGGACATCCCAC--GGAGGGGTGTAAAGCTGG--GCC

[B](http://genome.ucsc.edu/cgi-bin/hgTracks?db=musFur1&ct=&position=GL896969%3A6100183-6100279) [D](http://genome.ucsc.edu/cgi-bin/hgc?o=6100182&g=getDna&i=GL896969&c=GL896969&l=6100182&r=6100279&db=musFur1) Ferret CCTGGCTTCTCTCGTCCCCT--GGGCAGCTTGGTGGGCGTTCCGC--AG-GGGGTCCCAAGTGGG--GCC

[B](http://genome.ucsc.edu/cgi-bin/hgTracks?db=ailMel1&ct=&position=GL193710.1%3A135295-135392) [D](http://genome.ucsc.edu/cgi-bin/hgc?o=135294&g=getDna&i=GL193710.1&c=GL193710.1&l=135294&r=135392&db=ailMel1) Panda CCTGGCTTTTCTTGTCCACT--GGGCGGGCTGGTGGACGTTCCAT--GGAGGGGTCCAAAGCTGG--GCG

Pacific walrus ACTGGCCTCTTTTGTCCACT--GGGCGGGCTGGTGGACGTTCCGT--GGAGGGGTCCAAAGCTGG--GCC

Weddell seal ACTGGCCTCTCTTGTCCACT--GGGCGGGCTGGTGGACGTTCCGT--GGAGGGGTCCGAAGCTGG--GCC

Black flying-fox ACTGGCTTCTCTTGACCACT--TGGTGGGCTGGCGGACATTCCG---GGGGGCATTGGAAGCCGG--GCC

[B](http://genome.ucsc.edu/cgi-bin/hgTracks?db=pteVam1&ct=&position=scaffold_27084%3A3191-3287) [D](http://genome.ucsc.edu/cgi-bin/hgc?o=3190&g=getDna&i=scaffold_27084&c=scaffold_27084&l=3190&r=3287&db=pteVam1&hgSeq.revComp=on) Megabat ACTGGCTTCTCTTGACCACT--TGGTGGGCTGGCGGACGTTCCG---GGGGCCACCGGAAGCCGG--GCC

Big brown bat ACTGGCTCCTCTAGTCCACT--GGGTGGGCTGGTGGACACTCTGT--GGAGGGATCCAGGGCTGG--GCC

[B](http://genome.ucsc.edu/cgi-bin/hgTracks?db=triMan1&ct=&position=JH594719%3A2461558-2461619) [D](http://genome.ucsc.edu/cgi-bin/hgc?o=2461557&g=getDna&i=JH594719&c=JH594719&l=2461557&r=2461619&db=triMan1&hgSeq.revComp=on) Manatee AGG----CCAGCGGGCAGCG--CCGAGGGCAGTGGGA------------GGGATTCCGGGGGCTGGGGCC

[B](http://genome.ucsc.edu/cgi-bin/hgTracks?db=dasNov3&ct=&position=JH583290%3A23178-23282) [D](http://genome.ucsc.edu/cgi-bin/hgc?o=23177&g=getDna&i=JH583290&c=JH583290&l=23177&r=23282&db=dasNov3&hgSeq.revComp=on) Armadillo ACAGTCCCCTGCTGTCCACT--CTG**CAG**GCTGCTGGAC--CTCCT--GGAGGAGCCCACGCCTGAGGGCC

Cape elephant shrew ======================================================================

[B](http://genome.ucsc.edu/cgi-bin/hgTracks?db=mm10&ct=&position=chr7%3A145112604-145113522) [D](http://genome.ucsc.edu/cgi-bin/hgc?o=145112603&g=getDna&i=chr7&c=chr7&l=145112603&r=145113522&db=mm10&hgSeq.revComp=on) Mouse ======================================================================

Prairie vole ======================================================================

[B](http://genome.ucsc.edu/cgi-bin/hgTracks?db=rn5&ct=&position=chr1%3A225141922-225142822) [D](http://genome.ucsc.edu/cgi-bin/hgc?o=225141921&g=getDna&i=chr1&c=chr1&l=225141921&r=225142822&db=rn5&hgSeq.revComp=on) Rat ======================================================================

Cape golden mole ======================================================================

[B](http://genome.ucsc.edu/cgi-bin/hgTracks?db=criGri1&ct=&position=KE378474%3A1855202-1856134) [D](http://genome.ucsc.edu/cgi-bin/hgc?o=1855201&g=getDna&i=KE378474&c=KE378474&l=1855201&r=1856134&db=criGri1) Chinese hamster ======================================================================

Golden hamster ======================================================================

Aardvark ======================================================================

Lesser Egyptian jerboa ======================================================================

Brush-tailed rat ======================================================================

Chinchilla ======================================================================

[B](http://genome.ucsc.edu/cgi-bin/hgTracks?db=cavPor3&ct=&position=scaffold_42%3A9185303-9185540) [D](http://genome.ucsc.edu/cgi-bin/hgc?o=9185302&g=getDna&i=scaffold_42&c=scaffold_42&l=9185302&r=9185540&db=cavPor3) Guinea pig ======================================================================

White-throated sparrow ======================================================================

[B](http://genome.ucsc.edu/cgi-bin/hgTracks?db=geoFor1&ct=&position=JH739992%3A1483124-1483123) [D](http://genome.ucsc.edu/cgi-bin/hgc?o=1483123&g=getDna&i=JH739992&c=JH739992&l=1483123&r=1483123&db=geoFor1) Medium ground finch ======================================================================

Peregrine falcon ======================================================================

Saker falcon ======================================================================

Collared flycatcher ======================================================================

Tibetan ground jay ======================================================================

Mallard duck ======================================================================

[B](http://genome.ucsc.edu/cgi-bin/hgTracks?db=melUnd1&ct=&position=JH556606%3A2045680-2045679) [D](http://genome.ucsc.edu/cgi-bin/hgc?o=2045679&g=getDna&i=JH556606&c=JH556606&l=2045679&r=2045679&db=melUnd1) Budgerigar ======================================================================

Rock pigeon ======================================================================

[B](http://genome.ucsc.edu/cgi-bin/hgTracks?db=bosTau7&ct=&position=chrUn_JH121853%3A27622-27854) [D](http://genome.ucsc.edu/cgi-bin/hgc?o=27621&g=getDna&i=chrUn_JH121853&c=chrUn_JH121853&l=27621&r=27854&db=bosTau7) Cow ======================================================================

Domestic goat ======================================================================

[B](http://genome.ucsc.edu/cgi-bin/hgTracks?db=oviAri3&ct=&position=chr21%3A45885119-45885351) [D](http://genome.ucsc.edu/cgi-bin/hgc?o=45885118&g=getDna&i=chr21&c=chr21&l=45885118&r=45885351&db=oviAri3) Sheep ======================================================================

Tibetan antelope ======================================================================

[B](http://genome.ucsc.edu/cgi-bin/hgTracks?db=myoLuc2&ct=&position=GL429985%3A167075-167074) [D](http://genome.ucsc.edu/cgi-bin/hgc?o=167074&g=getDna&i=GL429985&c=GL429985&l=167074&r=167074&db=myoLuc2) Microbat ----------------------------------------------------------------------

David's myotis (bat) ======================================================================

[B](http://genome.ucsc.edu/cgi-bin/hgTracks?db=loxAfr3&ct=&position=scaffold_71%3A509092-509091) [D](http://genome.ucsc.edu/cgi-bin/hgc?o=509091&g=getDna&i=scaffold_71&c=scaffold_71&l=509091&r=509091&db=loxAfr3&hgSeq.revComp=on) Elephant ----------------------------------------------------------------------

[B](http://genome.ucsc.edu/cgi-bin/hgTracks?db=speTri2&ct=&position=JH393409%3A1184098-1185245) [D](http://genome.ucsc.edu/cgi-bin/hgc?o=1184097&g=getDna&i=JH393409&c=JH393409&l=1184097&r=1185245&db=speTri2&hgSeq.revComp=on) Squirrel ======================================================================

Chinese tree shrew ======================================================================

[B](http://genome.ucsc.edu/cgi-bin/hgTracks?db=hetGla2&ct=&position=JH602080%3A17761395-17762045) [D](http://genome.ucsc.edu/cgi-bin/hgc?o=17761394&g=getDna&i=JH602080&c=JH602080&l=17761394&r=17762045&db=hetGla2&hgSeq.revComp=on) Naked mole-rat ======================================================================

[B](http://genome.ucsc.edu/cgi-bin/hgTracks?db=ornAna1&ct=&position=chr3%3A7531224-7590302) [D](http://genome.ucsc.edu/cgi-bin/hgc?o=7531223&g=getDna&i=chr3&c=chr3&l=7531223&r=7590302&db=ornAna1) Platypus ======================================================================

[B](http://genome.ucsc.edu/cgi-bin/hgTracks?db=macEug2&ct=&position=GL105664%3A20690-59466) [D](http://genome.ucsc.edu/cgi-bin/hgc?o=20689&g=getDna&i=GL105664&c=GL105664&l=20689&r=59466&db=macEug2) Wallaby ======================================================================

Soft-shell Turtle ======================================================================

Green seaturtle ======================================================================

[B](http://genome.ucsc.edu/cgi-bin/hgTracks?db=melGal1&ct=&position=chr5%3A17376478-17407513) [D](http://genome.ucsc.edu/cgi-bin/hgc?o=17376477&g=getDna&i=chr5&c=chr5&l=17376477&r=17407513&db=melGal1) Turkey ======================================================================

[B](http://genome.ucsc.edu/cgi-bin/hgTracks?db=taeGut2&ct=&position=chr5%3A4972475-5001515) [D](http://genome.ucsc.edu/cgi-bin/hgc?o=4972474&g=getDna&i=chr5&c=chr5&l=4972474&r=5001515&db=taeGut2) Zebra finch ======================================================================

[B](http://genome.ucsc.edu/cgi-bin/hgTracks?db=sarHar1&ct=&position=chr6_GL864908_random%3A330350-372620) [D](http://genome.ucsc.edu/cgi-bin/hgc?o=330349&g=getDna&i=chr6_GL864908_random&c=chr6_GL864908_random&l=330349&r=372620&db=sarHar1) Tasmanian devil ======================================================================

[B](http://genome.ucsc.edu/cgi-bin/hgTracks?db=galGal4&ct=&position=chr5%3A16527834-16527833) [D](http://genome.ucsc.edu/cgi-bin/hgc?o=16527833&g=getDna&i=chr5&c=chr5&l=16527833&r=16527833&db=galGal4&hgSeq.revComp=on) Chicken ======================================================================

[B](http://genome.ucsc.edu/cgi-bin/hgTracks?db=allMis1&ct=&position=JH737694%3A164551-223805) [D](http://genome.ucsc.edu/cgi-bin/hgc?o=164550&g=getDna&i=JH737694&c=JH737694&l=164550&r=223805&db=allMis1) American alligator ======================================================================

[B](http://genome.ucsc.edu/cgi-bin/hgTracks?db=monDom5&ct=&position=chr5%3A304021248-304050406) [D](http://genome.ucsc.edu/cgi-bin/hgc?o=304021247&g=getDna&i=chr5&c=chr5&l=304021247&r=304050406&db=monDom5) Opossum ======================================================================

**(III)**

**Alignment block 44 of 70 in window, 69063214 - 69063402, 189 bps**

[B](http://genome.ucsc.edu/cgi-bin/hgTracks?db=hg19&ct=&position=chr11%3A69063214-69063402) [D](http://genome.ucsc.edu/cgi-bin/hgc?o=69063213&g=getDna&i=chr11&c=chr11&l=69063213&r=69063402&db=hg19) Human ------TGCTGCTGGAG----CTGGTGACCG-GGAGAGAAACAAGG--GAGAC--AAG--GGTGCCCAGA

[B](http://genome.ucsc.edu/cgi-bin/hgTracks?db=panTro4&ct=&position=chr11%3A67008677-67008865) [D](http://genome.ucsc.edu/cgi-bin/hgc?o=67008676&g=getDna&i=chr11&c=chr11&l=67008676&r=67008865&db=panTro4) Chimp ------TGCTGCTGGAA----CTGGTGACCG-GGAGAGAAACAAGG--GAGAC--AAG--GGTGTCCAGA

[B](http://genome.ucsc.edu/cgi-bin/hgTracks?db=gorGor3&ct=&position=chr11%3A66278631-66278819) [D](http://genome.ucsc.edu/cgi-bin/hgc?o=66278630&g=getDna&i=chr11&c=chr11&l=66278630&r=66278819&db=gorGor3) Gorilla ------TGCTGCTGGAG----CTGGTGACCG-GGAGAGAAACAAGG--GAGAC--AAG--GGTGTCCAGA

[B](http://genome.ucsc.edu/cgi-bin/hgTracks?db=ponAbe2&ct=&position=chr11%3A6739134-6739324) [D](http://genome.ucsc.edu/cgi-bin/hgc?o=6739133&g=getDna&i=chr11&c=chr11&l=6739133&r=6739324&db=ponAbe2&hgSeq.revComp=on) Orangutan ------TGCTGCCGGAG----CTGGTGACTG-GGAGAGAAACAAGGGAGAGAC--AAG--GGTGTCCAGA

[B](http://genome.ucsc.edu/cgi-bin/hgTracks?db=nomLeu3&ct=&position=chr4%3A89527968-89528156) [D](http://genome.ucsc.edu/cgi-bin/hgc?o=89527967&g=getDna&i=chr4&c=chr4&l=89527967&r=89528156&db=nomLeu3) Gibbon ------TGCTGCTGGAG----CTGATGACCG-GGAGAGAAACAAGG--GAGAC--AAG--GGTGTCCAGA

[B](http://genome.ucsc.edu/cgi-bin/hgTracks?db=rheMac3&ct=&position=chr14%3A5311896-5312070) [D](http://genome.ucsc.edu/cgi-bin/hgc?o=5311895&g=getDna&i=chr14&c=chr14&l=5311895&r=5312070&db=rheMac3&hgSeq.revComp=on) Rhesus ------TGCTGCTGGAG----CTAATGACCT-GGAGAGAAACAAGG--GAGAC--AAG--GGTGTCCAGA

Crab-eating macaque ------TGCTGCTGGAG----CTAATGACCT-GGAGAGAAACAAGG--GAGAC--AAG--GGTGTCCAGA

[B](http://genome.ucsc.edu/cgi-bin/hgTracks?db=papHam1&ct=&position=scaffold26046%3A4070-4244) [D](http://genome.ucsc.edu/cgi-bin/hgc?o=4069&g=getDna&i=scaffold26046&c=scaffold26046&l=4069&r=4244&db=papHam1) Baboon ------TGCTGCTGGAG----CTAATGACCT-GGAGAGAAACAAGG--GAGAC--AAG--GGTGTCCAGA

Green monkey ------TGCTGCTGGAG----CTAATGACCT-GGAGAGAAATAAGG--GAGAC--AAG--GGTGTCCAGA

[B](http://genome.ucsc.edu/cgi-bin/hgTracks?db=calJac3&ct=&position=chr11_GL285516_random%3A4068-4255) [D](http://genome.ucsc.edu/cgi-bin/hgc?o=4067&g=getDna&i=chr11_GL285516_random&c=chr11_GL285516_random&l=4067&r=4255&db=calJac3) Marmoset ------TGCTGCTGAAG----CTGATGACC--AGAGAGAAGCAAGG--GAGAC--AAG--CCTCTCCAGA

[B](http://genome.ucsc.edu/cgi-bin/hgTracks?db=saiBol1&ct=&position=JH378247%3A2100349-2100537) [D](http://genome.ucsc.edu/cgi-bin/hgc?o=2100348&g=getDna&i=JH378247&c=JH378247&l=2100348&r=2100537&db=saiBol1&hgSeq.revComp=on) Squirrel monkey ------TGCTGCTGAAG----CTAATGATC--AGAGAGAAGCAAGG--GAGAC--AAG--CCTGTCCAGA

[B](http://genome.ucsc.edu/cgi-bin/hgTracks?db=otoGar3&ct=&position=GL873653%3A1412860-1413059) [D](http://genome.ucsc.edu/cgi-bin/hgc?o=1412859&g=getDna&i=GL873653&c=GL873653&l=1412859&r=1413059&db=otoGar3&hgSeq.revComp=on) Bushbaby ------TGCTGCTGGAGTTCCCTCAGGAAAA-AGAAAGAAACAGCGGTGAAAT--GAG--TGTGTCCAGA

[B](http://genome.ucsc.edu/cgi-bin/hgTracks?db=cavPor3&ct=&position=scaffold_42%3A9185541-9185707) [D](http://genome.ucsc.edu/cgi-bin/hgc?o=9185540&g=getDna&i=scaffold_42&c=scaffold_42&l=9185540&r=9185707&db=cavPor3) Guinea pig ------TGCTGTTGGAG----CTCA-GAACA-AAAGAGAAATGAGA--GGGAT--ATGAAAGCATCCAAT

Chinchilla ------TGCTGCTGGAG----CTTG-GAACA-GAGGAGAAATGAGA--GGGAT--ATGAAGGTATCCAGT

Brush-tailed rat ------CACTGCTGGAG----CTTG-GAACA-GAAGAGAAATGAGA--GGGAT--ATGAGGGCATCCAGT

Pika ------GGACGCTGGGT----CCTG-GAGC---------CCTGAGG--GG----------ACCGTCCAGA

[B](http://genome.ucsc.edu/cgi-bin/hgTracks?db=susScr3&ct=&position=chr2%3A2576361-2576526) [D](http://genome.ucsc.edu/cgi-bin/hgc?o=2576360&g=getDna&i=chr2&c=chr2&l=2576360&r=2576526&db=susScr3&hgSeq.revComp=on) Pig ------GGCTGCTGGAG----CCGGA----A--------------------GA-------GGGGGCCAA-

[B](http://genome.ucsc.edu/cgi-bin/hgTracks?db=vicPac2&ct=&position=KB632549%3A2271073-2271252) [D](http://genome.ucsc.edu/cgi-bin/hgc?o=2271072&g=getDna&i=KB632549&c=KB632549&l=2271072&r=2271252&db=vicPac2) Alpaca ------TGCCGCTGGAG----CTGGG----GCAGGGACAGAAAGAG--AGGAA--GCT--GGAGGACGGG

Bactrian camel ------CGCCGCTGGAG----CTGGG----GCAGGGACAGAAAGAG--AGGAC--GCT--GGAGGACGGG

[B](http://genome.ucsc.edu/cgi-bin/hgTracks?db=turTru2&ct=&position=JH472550%3A248783-248952) [D](http://genome.ucsc.edu/cgi-bin/hgc?o=248782&g=getDna&i=JH472550&c=JH472550&l=248782&r=248952&db=turTru2) Dolphin ------TGCCCCTGGAG----CCGGG----A-GTGGACAGAGGGAG--AGGAA-------GGAGGACGA-

Killer whale ------TGCCCCTGGAG----CCGGG----G-GTGGACAGAGGGAG--AGGAA-------GGAGGACGA-

[B](http://genome.ucsc.edu/cgi-bin/hgTracks?db=equCab2&ct=&position=chr12%3A28435969-28436151) [D](http://genome.ucsc.edu/cgi-bin/hgc?o=28435968&g=getDna&i=chr12&c=chr12&l=28435968&r=28436151&db=equCab2) Horse ------TGCCACCAGAG----CTGGT-GCA--GGGGAGAAAGAGA---GGAAA--GAG--GGAGGCCAGG

[B](http://genome.ucsc.edu/cgi-bin/hgTracks?db=cerSim1&ct=&position=JH767824%3A4853475-4853654) [D](http://genome.ucsc.edu/cgi-bin/hgc?o=4853474&g=getDna&i=JH767824&c=JH767824&l=4853474&r=4853654&db=cerSim1&hgSeq.revComp=on) White rhinoceros ------TGCCACCAGAG----CCTGT-GCAG-GGGGAGAAAGAGGC--AGGGA--GAG--GGAGTCCAGG

[B](http://genome.ucsc.edu/cgi-bin/hgTracks?db=felCat5&ct=&position=chrD1%3A112914662-112914784) [D](http://genome.ucsc.edu/cgi-bin/hgc?o=112914661&g=getDna&i=chrD1&c=chrD1&l=112914661&r=112914784&db=felCat5) Cat ------GGCCACT---------------------GGAGAAACTGGG--AGGAA--GAG--GGAGTCCAGG

[B](http://genome.ucsc.edu/cgi-bin/hgTracks?db=canFam3&ct=&position=chr18%3A48781990-48782115) [D](http://genome.ucsc.edu/cgi-bin/hgc?o=48781989&g=getDna&i=chr18&c=chr18&l=48781989&r=48782115&db=canFam3&hgSeq.revComp=on) Dog ------CGCCACTGGGC----ATGGGTGCAG-AGGGAGAAACTGGG--AGGAA--GAG--GAG-------

[B](http://genome.ucsc.edu/cgi-bin/hgTracks?db=musFur1&ct=&position=GL896969%3A6100322-6100496) [D](http://genome.ucsc.edu/cgi-bin/hgc?o=6100321&g=getDna&i=GL896969&c=GL896969&l=6100321&r=6100496&db=musFur1) Ferret ------TGCTGCTAGAG----CCAGG-GCAG-ACGGTGAACCCGGG--TGGAA--GAG--GGAATCCGGG

[B](http://genome.ucsc.edu/cgi-bin/hgTracks?db=ailMel1&ct=&position=GL193710.1%3A135435-135609) [D](http://genome.ucsc.edu/cgi-bin/hgc?o=135434&g=getDna&i=GL193710.1&c=GL193710.1&l=135434&r=135609&db=ailMel1) Panda ------TGCCACTGGAG----CCAGT-GCAG-AGGGAGAAACCTGG--AGGAC--GAG--GGAGTCTGGG

Pacific walrus ------TGCCACTGGAG----CCAGG-GCAG-AGGGAGAACCTGGG--AGGAA--GAG--GGAGTGTGGG

Weddell seal ------TGCCACTGGAG----CC-------G-AGGGAGAACCTGGG--AGGAA--GAG--GGAGTCTGGG

[B](http://genome.ucsc.edu/cgi-bin/hgTracks?db=pteVam1&ct=&position=scaffold_27084%3A3098-3164) [D](http://genome.ucsc.edu/cgi-bin/hgc?o=3097&g=getDna&i=scaffold_27084&c=scaffold_27084&l=3097&r=3164&db=pteVam1&hgSeq.revComp=on) Megabat ------------------------------G-CGAAAGCGCCTCGT--AGGGT--GAC--GGCTGCT---

Big brown bat ------------------------------G-GCAAAGGGCCTCTC--AGGGT--GAT--GGCTGCTG--

[B](http://genome.ucsc.edu/cgi-bin/hgTracks?db=triMan1&ct=&position=JH594719%3A2461421-2461544) [D](http://genome.ucsc.edu/cgi-bin/hgc?o=2461420&g=getDna&i=JH594719&c=JH594719&l=2461420&r=2461544&db=triMan1&hgSeq.revComp=on) Manatee -------ACTCTCGGTG----TG--------------------------------AAT--GTGGGATAG-

[B](http://genome.ucsc.edu/cgi-bin/hgTracks?db=dasNov3&ct=&position=JH583290%3A22964-23148) [D](http://genome.ucsc.edu/cgi-bin/hgc?o=22963&g=getDna&i=JH583290&c=JH583290&l=22963&r=23148&db=dasNov3&hgSeq.revComp=on) Armadillo agagccTGCTGCCGGAG----CGTGTGAAGA-GGAGGGGAGCGAAG--GAGAAGAAAT--GTGGACCAGC

Cape elephant shrew ======================================================================

[B](http://genome.ucsc.edu/cgi-bin/hgTracks?db=mm10&ct=&position=chr7%3A145112604-145113522) [D](http://genome.ucsc.edu/cgi-bin/hgc?o=145112603&g=getDna&i=chr7&c=chr7&l=145112603&r=145113522&db=mm10&hgSeq.revComp=on) Mouse ======================================================================

Prairie vole ======================================================================

[B](http://genome.ucsc.edu/cgi-bin/hgTracks?db=rn5&ct=&position=chr1%3A225141922-225142822) [D](http://genome.ucsc.edu/cgi-bin/hgc?o=225141921&g=getDna&i=chr1&c=chr1&l=225141921&r=225142822&db=rn5&hgSeq.revComp=on) Rat ======================================================================

Cape golden mole ======================================================================

[B](http://genome.ucsc.edu/cgi-bin/hgTracks?db=criGri1&ct=&position=KE378474%3A1855202-1856134) [D](http://genome.ucsc.edu/cgi-bin/hgc?o=1855201&g=getDna&i=KE378474&c=KE378474&l=1855201&r=1856134&db=criGri1) Chinese hamster ======================================================================

Golden hamster ======================================================================

Aardvark ======================================================================

Black flying-fox ======================================================================

Lesser Egyptian jerboa ======================================================================

White-throated sparrow ======================================================================

[B](http://genome.ucsc.edu/cgi-bin/hgTracks?db=geoFor1&ct=&position=JH739992%3A1483124-1483123) [D](http://genome.ucsc.edu/cgi-bin/hgc?o=1483123&g=getDna&i=JH739992&c=JH739992&l=1483123&r=1483123&db=geoFor1) Medium ground finch ======================================================================

Peregrine falcon ======================================================================

Saker falcon ======================================================================

Collared flycatcher ======================================================================

Tibetan ground jay ======================================================================

Mallard duck ======================================================================

[B](http://genome.ucsc.edu/cgi-bin/hgTracks?db=melUnd1&ct=&position=JH556606%3A2045680-2045679) [D](http://genome.ucsc.edu/cgi-bin/hgc?o=2045679&g=getDna&i=JH556606&c=JH556606&l=2045679&r=2045679&db=melUnd1) Budgerigar ======================================================================

Rock pigeon ======================================================================

[B](http://genome.ucsc.edu/cgi-bin/hgTracks?db=bosTau7&ct=&position=chrUn_JH121853%3A27622-27854) [D](http://genome.ucsc.edu/cgi-bin/hgc?o=27621&g=getDna&i=chrUn_JH121853&c=chrUn_JH121853&l=27621&r=27854&db=bosTau7) Cow ======================================================================

Domestic goat ======================================================================

[B](http://genome.ucsc.edu/cgi-bin/hgTracks?db=oviAri3&ct=&position=chr21%3A45885119-45885351) [D](http://genome.ucsc.edu/cgi-bin/hgc?o=45885118&g=getDna&i=chr21&c=chr21&l=45885118&r=45885351&db=oviAri3) Sheep ======================================================================

Tibetan antelope ======================================================================

[B](http://genome.ucsc.edu/cgi-bin/hgTracks?db=myoLuc2&ct=&position=GL429985%3A167075-167074) [D](http://genome.ucsc.edu/cgi-bin/hgc?o=167074&g=getDna&i=GL429985&c=GL429985&l=167074&r=167074&db=myoLuc2) Microbat ----------------------------------------------------------------------

David's myotis (bat) ======================================================================

[B](http://genome.ucsc.edu/cgi-bin/hgTracks?db=loxAfr3&ct=&position=scaffold_71%3A509092-509091) [D](http://genome.ucsc.edu/cgi-bin/hgc?o=509091&g=getDna&i=scaffold_71&c=scaffold_71&l=509091&r=509091&db=loxAfr3&hgSeq.revComp=on) Elephant ----------------------------------------------------------------------

[B](http://genome.ucsc.edu/cgi-bin/hgTracks?db=speTri2&ct=&position=JH393409%3A1184098-1185245) [D](http://genome.ucsc.edu/cgi-bin/hgc?o=1184097&g=getDna&i=JH393409&c=JH393409&l=1184097&r=1185245&db=speTri2&hgSeq.revComp=on) Squirrel ======================================================================

Chinese tree shrew ======================================================================

[B](http://genome.ucsc.edu/cgi-bin/hgTracks?db=hetGla2&ct=&position=JH602080%3A17761395-17762045) [D](http://genome.ucsc.edu/cgi-bin/hgc?o=17761394&g=getDna&i=JH602080&c=JH602080&l=17761394&r=17762045&db=hetGla2&hgSeq.revComp=on) Naked mole-rat ======================================================================

[B](http://genome.ucsc.edu/cgi-bin/hgTracks?db=ornAna1&ct=&position=chr3%3A7531224-7590302) [D](http://genome.ucsc.edu/cgi-bin/hgc?o=7531223&g=getDna&i=chr3&c=chr3&l=7531223&r=7590302&db=ornAna1) Platypus ======================================================================

[B](http://genome.ucsc.edu/cgi-bin/hgTracks?db=macEug2&ct=&position=GL105664%3A20690-59466) [D](http://genome.ucsc.edu/cgi-bin/hgc?o=20689&g=getDna&i=GL105664&c=GL105664&l=20689&r=59466&db=macEug2) Wallaby ======================================================================

Soft-shell Turtle ======================================================================

Green seaturtle ======================================================================

[B](http://genome.ucsc.edu/cgi-bin/hgTracks?db=melGal1&ct=&position=chr5%3A17376478-17407513) [D](http://genome.ucsc.edu/cgi-bin/hgc?o=17376477&g=getDna&i=chr5&c=chr5&l=17376477&r=17407513&db=melGal1) Turkey ======================================================================

[B](http://genome.ucsc.edu/cgi-bin/hgTracks?db=taeGut2&ct=&position=chr5%3A4972475-5001515) [D](http://genome.ucsc.edu/cgi-bin/hgc?o=4972474&g=getDna&i=chr5&c=chr5&l=4972474&r=5001515&db=taeGut2) Zebra finch ======================================================================

[B](http://genome.ucsc.edu/cgi-bin/hgTracks?db=sarHar1&ct=&position=chr6_GL864908_random%3A330350-372620) [D](http://genome.ucsc.edu/cgi-bin/hgc?o=330349&g=getDna&i=chr6_GL864908_random&c=chr6_GL864908_random&l=330349&r=372620&db=sarHar1) Tasmanian devil ======================================================================

[B](http://genome.ucsc.edu/cgi-bin/hgTracks?db=galGal4&ct=&position=chr5%3A16527834-16527833) [D](http://genome.ucsc.edu/cgi-bin/hgc?o=16527833&g=getDna&i=chr5&c=chr5&l=16527833&r=16527833&db=galGal4&hgSeq.revComp=on) Chicken ======================================================================

[B](http://genome.ucsc.edu/cgi-bin/hgTracks?db=allMis1&ct=&position=JH737694%3A164551-223805) [D](http://genome.ucsc.edu/cgi-bin/hgc?o=164550&g=getDna&i=JH737694&c=JH737694&l=164550&r=223805&db=allMis1) American alligator ======================================================================

[B](http://genome.ucsc.edu/cgi-bin/hgTracks?db=monDom5&ct=&position=chr5%3A304021248-304050406) [D](http://genome.ucsc.edu/cgi-bin/hgc?o=304021247&g=getDna&i=chr5&c=chr5&l=304021247&r=304050406&db=monDom5) Opossum ======================================================================

**(IV)**

Human GTGGACGTG-TCCCGGG----CCAGGAGGGTCAC--------AGATGCACCACAAGGCA--------CTC

Chimp GTGGACGTG-TCCCGGG----CCAGGAGGGTCAC--------AGATGCACCACAAGGCA--------CTC

Gorilla GTGGACGTG-TCCCGGG----CCAGGAGGGTCAC--------AGATGCACCACAAGACA--------CTC

Orangutan ACGGACGTG-TCCCGGG----CCAGGAGGGTCAC--------AGATGCACCACCAGGTA--------CTC

Gibbon GTGGACATG-TCCTGGG----CCAGGAGGGTCAC--------AGATGCGCCACTAGGCA--------CTC

Rhesus GTGGATGTG-TCCCAGG----CCA**----------**--------**----**GCGCCACCAGGCA--------CTC

Crab-eating macaque GTGGATGTG-TCCCAGG----CCA**----------**--------**----**GCGCCACCAGGCA--------CTC

Baboon GTGGATGTG-TCCCGGG----CCA**----------**--------**----**GCGCCACCAGGCA--------CTC

Green monkey GTGGATGTG-TCCCGGG----CCA**----------**--------**----**GCGCCACCAGGCA--------CTC

Marmoset ACAGACCTG-CCCCGGG----CCAGGCAGGTCAC--------AGATGCACCACCAGGCA--------CTC

Squirrel monkey GCAGACCTGCCCCCGGG----CCAGGCGGGTCAC--------AGATGCGCCACCAGGCA--------CTC

Bushbaby ACGGATGTG-CCATAGC----CCAGGCAGGTCAT--------GGATATGCCACCA---------------

Guinea pig GTGGACATG-CCATGGG----CTG-GGAGGTCAG--------GGAGCCACCAAGAA--------------

Chinchilla GTGGACATG-TCATGGG----CCA-GAAGGCTAG--------GGAGGTACCAAGAT--------------

Brush-tailed rat GCTGACATG-CCACGGG----CCA-TGCAGCCAC--------GGGGGCGCCAAGAA--------------

Pika GGGGCCACA-CCACAGGCGCTCCG-TGGGGCCTG--------GAG------CAGAA--------------

Pig CTGGCCTCA-CCTCGGG----TGT-----GCCACA-------GGATG--CCATGGGGGT--------CCC

Alpaca ATGGGCACG-CCTCGGG----AGG-----GCCAT--------GGATG--CCACCA---------------

Bactrian camel ATGGGCACG-CCTCGGG----AGG-----GCCAT--------GGATG--CCACCA---------------

Dolphin ACGGACACG-CCTCG-------GG-----GCCAC--------GGATG--CCACCGTGAG--------CCC

Killer whale ACGGACACG-CCTCG-------GG-----GCCAC--------GGATG--CCACCGTGAG--------CCC

Horse ATGGACATG-CCTCGGG-----GG-----GCCAC---------GATG--CTGCCAGGAGGCCCCCCCCCC

White rhinoceros ACGGACACG-CCTCAGG-----GG-----GCCAC---------GATG--CCACCAGGAG--------CCC

Cat ------------TCCGG----AGG-----GCCAC--------GGATG--CCACCAGGCG--------CCC

Dog ------------TCGGG----CCG-----GCCAC--------GGAGG---CACCGGGAG--------CCC

Ferret ACCGCCGTG-CCTCAGG----CTG-----GCCCC--------GGAGG--CCACCAGGAG--------CCC

Panda ATAGCC-TG-CCTTGGG----CTG-----GCCAC--------GGATG--CCACCAGGAG--------CCT

Pacific walrus ACAGCCACG-CCTTGGG----CCA-----GCCAC--------GGATG--CCACCAGGAG--------CCC

Weddell seal ACAGCCACG-CCTCGGG----CCA-----GCCAC--------GGATG--CCACCAGGAG--------CCC

Megabat -----------------------------GC------------------------GGAG--------CCA

Big brown bat CCGGACACA-CCTCCGA----CAG-----GCCAC--------CGA-----CACCAGGAG--------CCC

Manatee ----------TCTGGGG----GTC-----GTGA---------GGAGA--GGTGAACAGA--------CTC

Armadillo ----------CCACGGG----CTG-----ATGACGGAGGCCGGGCGC--GCAGCACGGG--------CTC

Cape elephant shrew ======================================================================

Mouse ======================================================================

Prairie vole ======================================================================

Rat ======================================================================

Cape golden mole ======================================================================

Chinese hamster ======================================================================

Golden hamster ======================================================================

Aardvark ======================================================================

Black flying-fox ======================================================================

Lesser Egyptian jerboa ======================================================================

White-throated sparrow ======================================================================

Medium ground finch ======================================================================

Peregrine falcon ======================================================================

Saker falcon ======================================================================

Collared flycatcher ======================================================================

Tibetan ground jay ======================================================================

Mallard duck ======================================================================

Budgerigar ======================================================================

Rock pigeon ======================================================================

Cow ======================================================================

Domestic goat ======================================================================

Sheep ======================================================================

Tibetan antelope ======================================================================

Microbat ----------------------------------------------------------------------

David's myotis (bat) ======================================================================

Elephant ----------------------------------------------------------------------

Squirrel ======================================================================

Chinese tree shrew ======================================================================

Naked mole-rat ======================================================================

Platypus ======================================================================

Wallaby ======================================================================

Soft-shell Turtle ======================================================================

Green seaturtle ======================================================================

Turkey ======================================================================

Zebra finch ======================================================================

Tasmanian devil ======================================================================

Chicken ======================================================================

American alligator ======================================================================

Opossum ======================================================================

Human TGT-GT-GGCAC-TG-GGAACAGGAATTCTGGGAGTCA--G--TCTGCAAGGGT---GGTGG--------

Chimp TGT-GT-GGCAC-TG-GGAACAGGAATTCTGGGAGTCA--G--TCTGCAAGGGC---GGTGG--------

Gorilla TGT-GT-GGCAC-TG-GGAACAGGAATTCTGGGAGTCA--G--TCTGCAAGGGC---GGTGG--------

Orangutan TGT-GT-GGCAC-TG-GGAACAGGAATTCTGGGAGTCA--G--TCTGCAAGGGC---GATGG--------

Gibbon TGT-GT-GGCAC-TG-GGAACAGGAATTCTGGGAGTCA--G--TCTTCAAGGGC---GGTGG--------

Rhesus TGT-GT-GGCAC-TG-GGAACAGGAATTC**TGA**GAGTTA--G--TCTGTAAGGGT---GGCGG--------

Crab-eating macaque TGT-GT-GGCAC-TG-GGAACAGGAATTC**TGA**GAGTTA--G--TCTGTAAGGGT---GGCGG--------

Baboon TGT-GT-GGCAC-TG-GGAACAGGAATTC**TGA**GAGTTA--G--TCTGTAAGGGT---GGCGG--------

Green monkey TGT-GT-GGCAC-TG-GGAACAGGAATTC**TGA**GAGTCA--G--TCTGTAAGGGT---GGCGG--------

Marmoset TGT-GT-GGCAC-TG-GGCACAGGAATTCTGGAATTCA--A--TCTGCAAGGGT---GGTAG--------

Squirrel monkey TGT-GT-GGCAC-TG-GGCACAGGAATTCTGGAATTCA--G--TCTGCAAGGGT---GGTGG--------

Bushbaby --T-GT-GGCGC-TGAGAAAAAAGGATTCTGGGAGTCAGTG--TGTGCAGGGGT---GGCAGGTGCCAGG

Guinea pig CGT-GT-TACTC-TG-GGACAGGGC---------------A--CTTGCAGGACA---GCCTG--------

Chinchilla TGC-GT-TGTTC-TG-GGACAGGGC---------------A--CCCACAGGGTA---GCCAG--------

Brush-tailed rat CGC-TT-TGCTC-TG-GGATAGGGC---------------A--CCTGCAGGGTG---GCCAG--------

Pika GGC-AT-CCCCAGCG-GGATGCAGC---------------G--GGTGCTGG------GCCAA--------

Pig CCT----GGGAC-GA-GATGGAGGGGTTCTGGGACACA--GCTTTTGCGGGGGG---GG-GGACCCCGGG

Alpaca -------GGGAT-GA-AATCGAGGGATT-TGGGACATG--ACTTCTGCGAGGGT---GGTTGGTGCCAGG

Bactrian camel -------GGGAT-GA-GATCGAGGGATT-TGGGACGTG--ACTTCTGCGAGGGT---GGTTGGTGCCGGG

Dolphin CGC-GT-GGGAT-GA-GACGCAGGGATTGTGGGACACG--GC-TCTGCGAGGGT---GGCGGGCGCCAGG

Killer whale CGC-GT-GGGAT-GA-GACGCAGGGATTCTGGGACACG--GC-TCTGCGAGGGT---GGCGGGCGCCAGG

Horse CG----GGAGTC-AA-GAACAAAGGATTCTGGGGAACG--GCTTATGCAGGGCG----------------

White rhinoceros CGG-GTGGAGTC-TA-GAACGAAGGATTCTGGGCAATG--GCTTCTGGGGGGCG----------------

Cat CTC-GTGGAGTC-AG-GGACAAGGGGTTCTGGGAAACG--G-----------------------------

Dog CGC-GTGGAGTC-AG-GGACGAGGGATTCTGGG-CACG--GCTTCTGCGAGCA-----------------

Ferret CGC-GTAGGGTC-AG-GGACAAGGGAATCTGGGAAACT--GCTTCTGCAAGCG-----------------

Panda TGC-GTGGAGTC-AG-GGACAAGGGATTCTGGGAAACG--GCTTCTGCAAGCA-----------------

Pacific walrus CGC-GTGGAGTC-AG-GGACAAGGGATTCTGGGAAACG--GCTTCTGCAAGCA-----------------

Weddell seal CGC-GTGGAGTC-AG-GGACAAGGGATTCTGGGAAACG--GCTTCTGCAAGTA-----------------

Megabat TGCAGGGGAGGA-GA-G--------------------------------AGGGG----------------

Big brown bat CGC-GTGGAGTC-GA-GCCCCAGTGTTTCTGGGAACCG--GCTGCTGCCAGGGT----------------

Manatee CAG-AA-GCCTC-TG-GAAG------CCTCTGGAAGCT--GCGGAGGATGGTGCCCAGGCAT--------

Armadillo CAC-GG-GCGCC-AG-GAAGAAAGGATTCTGGGAAACG--GCTCCTGTGGGTGCCAGGGCCA--------

Cape elephant shrew ======================================================================

Mouse ======================================================================

Prairie vole ======================================================================

Rat ======================================================================

Cape golden mole ======================================================================

Chinese hamster ======================================================================

Golden hamster ======================================================================

Aardvark ======================================================================

Black flying-fox ======================================================================

Lesser Egyptian jerboa ======================================================================

White-throated sparrow ======================================================================

Medium ground finch ======================================================================

Peregrine falcon ======================================================================

Saker falcon ======================================================================

Collared flycatcher ======================================================================

Tibetan ground jay ======================================================================

Mallard duck ======================================================================

Budgerigar ======================================================================

Rock pigeon ======================================================================

Cow ======================================================================

Domestic goat ======================================================================

Sheep ======================================================================

Tibetan antelope ======================================================================

Microbat ----------------------------------------------------------------------

David's myotis (bat) ======================================================================

Elephant ----------------------------------------------------------------------

Squirrel ======================================================================

Chinese tree shrew ======================================================================

Naked mole-rat ======================================================================

Platypus ======================================================================

Wallaby ======================================================================

Soft-shell Turtle ======================================================================

Green seaturtle ======================================================================

Turkey ======================================================================

Zebra finch ======================================================================

Tasmanian devil ======================================================================

Chicken ======================================================================

American alligator ======================================================================

Opossum ======================================================================

**Supplementary Data Set 2A**

**RepeatMasker annotation:** “The query species was assumed to be **homo sapiens**

RepeatMasker version open-4.0.5, **default mode**

Run with blastp version 3.0SE-AB [2009-10-30] [linux26-x64-I32LPF64 2009-10-30T17:06:09]_RepBase Update 20140131, RM database version 20140131”.

**RepeatMasker Matrix used: Based on GC level query.**

**position in query- -position in repeat-**

**% % % query C matching repeat (left) end begin linkage**

**+ score div. del. ins. sequence begin end (left) + repeat class/family begin end (left) id/graphic**

[+](javascript:;) **435 32.8 6.1 0.0 MYEOV 351 579 (2554) C** [**L2**](http://www.repeatmasker.org/cgi-bin/ViewRepeat?id=L2)  **LINE/L2 (511) 2908 2666 1**

**ANNOTATION EVIDENCE:**

435 32.75 6.11 0.00 MYEOV 351 579 2517 C [**L2**](http://www.repeatmasker.org/cgi-bin/ViewRepeat?id=L2)  LINE/L2 2666 2908 174

435 32.75 6.11 0.00 MYEOV 351 579 (2517) C L2#LINE/L2 (174) 2908 2666 m_b1s601i0

MYEOV 351 GCCTT-TGGGCT-CAGTGAAGAGTCTGGAGTTTATCTGGAGTGAGGTGGC 398

- i i- v i i v i vv iiiv i v

C L2#LINE/L2 2908 GCCTTGTAGGCCACTGTAAGGACTTTGGCTTTTACTCTGAGTGAGATGGG 2859

MYEOV 399 CGGTTCTTGGTGGGATCTGAGC**AGGTAAGA**AGCAGGGTCTTTCTTATGTT 448

vi iivi v v i ivi vii v i vv

C L2#LINE/L2 2858 AAGCCACTGGAGGGTTTTGAGC**AGAGGAGT**GACATGATCTGACTTATGTT 2809

MYEOV 449 TTAAGGAAAGCCCTGTGGCTGCTGGGTGGGGGACCAGCTGGAGGGG--CA 496

iiii v v v v v i i ivii ? --

C L2#LINE/L2 2808 TTAAAAGGATCACTCTGGCTGCTGTGTTGAGAATAGACTGNAGGGGGGCA 2759

MYEOV 497 GGAG-GGCAGCAGG-TGACTCATTACAAGGCTGGGGCCATCATCCTG-TG 543

i i - v -v ivi vi ivv vi v v -i

C L2#LINE/L2 2758 AGGGTGGAAGCAGGGAGACCAGTTAGGAGGCTATTGCAGTAATCCAGGCG 2709

MYEOV 544 AGAGCTG-----GGCATG-ACCAGTGCAG-GTCAGTGGGGTTG 579

v ----- v - v ii - v i v

C L2#LINE/L2 2708 AGAGATGATGGTGGCTTGGACCAGGGTGGTGGCAGTGGAGGTG 2666

Matrix = 25p53g.matrix

Kimura (with divCpGMod) = 42.61

Transitions / transversions = 1.11 (39/35)

Gap_init rate = 0.04 (9 / 228), avg. gap size = 1.56 (14 / 9)

[+](javascript:;) **250 34.0 43.3 0.0 MYEOV 1081 1297 (1836) +** [**L2a**](http://www.repeatmasker.org/cgi-bin/ViewRepeat?id=L2a)  **LINE/L2 2779 3089 (337) 2**

**ANNOTATION EVIDENCE:**

194 38.69 4.38 0.00 MYEOV 1081 1217 1879 + [**L2**](http://www.repeatmasker.org/cgi-bin/ViewRepeat?id=L2)  LINE/L2 2779 2921 161

194 38.69 4.38 0.00 MYEOV 1081 1217 (1879) L2#LINE/L2 2779 2921 (161) m_b1s601i1

MYEOV 1081 TCCATTCAGGAGCAGGAAAGTTCATCTCAGACCCTAAATCCAGCCACGTC 1130

v iv v vi iv v v i i i v v i viii i

L2#LINE/L2 2779 TCAACACAGCAGCCAGAGTGATCCTTTTAAAACATAAGTCAGATCATGTC 2828

MYEOV 1131 ATGCCAC-GCTTAACACC-TCTAACAA**CTTCCCCTGGCACTTAGG**ACAGC 1178

iv v - i v - i iiii v vv i iii iv

L2#LINE/L2 2829 ACTCCTCTGCTCAAAACCCTCCAGTGG**CTTCCCATCTCACTCAGA**GTAAA 2878

MYEOV 1179 -GTCTGGCTCCTTCCCTCGGCT--C**ATG**GCCCT-CAGAATCTG 1217

- i viiv v vvi i-- v - vi

L2#LINE/L2 2879 AGCCAAAGTCCTTACAGTGGCCTAC**AAG**GCCCTACATGATCTG 2921

Matrix = 25p53g.matrix

Kimura (with divCpGMod) = 56.42

Transitions / transversions = 1.21 (29/24)

Gap_init rate = 0.04 (5 / 136), avg. gap size = 1.20 (6 / 5)

250 26.51 7.23 0.00 MYEOV 1215 1297 1799 + [**L2a_3end**](http://www.repeatmasker.org/cgi-bin/ViewRepeat?id=L2a_3end) LINE/L2 92 180 337

250 26.51 7.23 0.00 MYEOV 1215 1297 (1799) L2a_3end#LINE/L2 92 180 (337) m_b1s601i2

MYEOV 1215 CTGCGTCACATACA**CCCCAG---CTCTCCCGAT---AGG**TCTCTGCACTC 1258

ivi v iv v --- v vi---i i i i

L2a_3end#LINE 92 CTGTTCCTCGAACA**CGCCAGGCACGCTCCCGCCTCAGGG**CCTTTGCACTT 141

MYEOV 1259 GCTGTTGCCTCTGCCTGGAACAGTCTCCCTCCTGGTGTC 1297

v iv i i v i i

L2a_3end#LINE 142 GCTGTTCCCTCTGCCTGGAACGCTCTTCCCCCAGATATC 180

Matrix = 25p53g.matrix

Kimura (with divCpGMod) = 29.88

Transitions / transversions = 1.44 (13/9)

Gap_init rate = 0.02 (2 / 82), avg. gap size = 3.00 (6 / 2)

[+](javascript:;) **27 5.5 2.6 0.0 MYEOV 2180 2217 (916) +** [**(CTC)n**](http://www.repeatmasker.org/cgi-bin/ViewRepeat?id=(CTC)n)  **Simple_repeat 1 39 (0) 3**

[+](javascript:;) **592 26.7 8.7 0.0 MYEOV 2218 2378 (755) C** [**MIR**](http://www.repeatmasker.org/cgi-bin/ViewRepeat?id=MIR)  **SINE/MIR (1) 261 87 4**

**ANNOTATION EVIDENCE:**

592 26.71 8.70 0.00 MYEOV 2218 2378 755 C [**MIR**](http://www.repeatmasker.org/cgi-bin/ViewRepeat?id=MIR)  SINE/MIR 87 261 1

592 26.71 8.70 0.00 MYEOV 2218 2378 (755) C MIR#SINE/MIR (1) 261 87 m_b1s601i3

MYEOV 2218 ATCATCATCCTCACTTGT**TGA**GGACGTCCTGTGTGCCA------------ 2255

v v i vv i i vi v v i ------------

C MIR#SINE/MIR 261 ATAATAACCAACATTTAT**TGA**GCGCTTACTATGTGCCAGGCACTGTTCTA 212

MYEOV 2256 AGTGGTTTATATGCCCAGCCTCATTTAATCCTCAGAATGACTCCATGAGG 2305

i v i iviviv v ii i i

C MIR#SINE/MIR 211 AGCGCTTTACATGTATTAACTCATTTAATCCTCACAACAACCCTATGAGG 162

MYEOV 2306 TAGCTACTAAAACC--CCCCACTTAACAGATGAGGAAACTGAGGCCTAGA 2353

v vv ii-- i v vi

C MIR#SINE/MIR 161 TAGGTACTATTATTATCCCCATTTTACAGATGAGGAAACTGAGGCACAGA 112

MYEOV 2354 GAAGCTCAACAAGTTGCCTAAGTTC 2378

i i v ii v i v

C MIR#SINE/MIR 111 GAGGTTAAGTAACTTGCCCAAGGTC 87

Matrix = 25p53g.matrix

Kimura (with divCpGMod) = 31.66

Transitions / transversions = 1.15 (23/20)

Gap_init rate = 0.01 (2 / 160), avg. gap size = 7.00 (14 / 2)

[+](javascript:;) **216 27.7 3.1 0.0 MYEOV 2447 2511 (622) C** [**L2a**](http://www.repeatmasker.org/cgi-bin/ViewRepeat?id=L2a)  **LINE/L2 (1) 3425 3359 5**

[+](javascript:;) **288 29.6 5.2 0.8 MYEOV 2600 2715 (418) +** [**MER117**](http://www.repeatmasker.org/cgi-bin/ViewRepeat?id=MER117)  **DNA/hAT-Charlie 45 165 (32) 6**

[+](javascript:;) **194 18.6 2.5 2.2 MYEOV 2815 2870 (263) C** [**MIRb**](http://www.repeatmasker.org/cgi-bin/ViewRepeat?id=MIRb)  **SINE/MIR (66) 164 108 7**

[+](javascript:;) **248 26.8 1.3 2.2 MYEOV 2907 3053 (80) +** [**L4_A_Mam**](http://www.repeatmasker.org/cgi-bin/ViewRepeat?id=L4_A_Mam) **LINE/RTE-X 4690 4781 (209) 8**

**Supplementary Data Set 2B**

**RepeatMasker annotation:** “The query species was assumed to be **homo sapiens**

RepeatMasker version open-4.0.5, **sensitive mode**

Run with cross_match version 1.080812_RepBase Update 20140131, RM database version 20140131”.

**RepeatMasker Matrix used: Based on GC level query.**

**position in query- -position in repeat-**

**% % % query C matching repeat (left) end begin linkage**

**+ score div. del. ins. sequence begin end (left) + repeat class/family begin end (left) id/graphic**

[+](javascript:;) **435 32.8 5.8 0.0 MYEOV 351 579 (2554) C** [**L2**](http://www.repeatmasker.org/cgi-bin/ViewRepeat?id=L2)  **LINE/L2 (511) 2908 2666 1**

**ANNOTATION EVIDENCE:**

435 32.75 5.76 0.00 MYEOV 351 579 2517 C [**L2**](http://www.repeatmasker.org/cgi-bin/ViewRepeat?id=L2)  LINE/L2 2666 2908 174

435 32.75 5.76 0.00 MYEOV 351 579 (2517) C L2#LINE/L2 (174) 2908 2666 m_b1s601i0

MYEOV 351 GCCTT-TGGGCT-CAGTGAAGAGTCTGGAGTTTATCTGGAGTGAGGTGGC 398

- i i- v i i v i vv iiiv i v

C L2#LINE/L2 2908 GCCTTGTAGGCCACTGTAAGGACTTTGGCTTTTACTCTGAGTGAGATGGG 2859

MYEOV 399 CGGTTCTTGGTGGGATCTGAGC**AGGTAAGA**AGCAGGGTCTTTCTTATGTT 448

vi iivi v v i ivi vii v i vv

C L2#LINE/L2 2858 AAGCCACTGGAGGGTTTTGAGC**AGAGGAGT**GACATGATCTGACTTATGTT 2809

MYEOV 449 TTAAGGAAAGCCCTGTGGCTGCTGGGTGGGGGACCAGCTGGA--GGGGCA 496

iiii v v v v v i i ivii ? --

C L2#LINE/L2 2808 TTAAAAGGATCACTCTGGCTGCTGTGTTGAGAATAGACTGNAGGGGGGCA 2759

MYEOV 497 GGAG-GGCAGCA-GGTGACTCATTACAAGGCTGGGGCCATCATCCT-GTG 543

i i - v - v ivi vi ivv vi v v- i

C L2#LINE/L2 2758 AGGGTGGAAGCAGGGAGACCAGTTAGGAGGCTATTGCAGTAATCCAGGCG 2709

MYEOV 544 AGAGCTG-----GGCAT-GACCAGTGCAG-GTCAGTGGGGTTG 579

v ----- v - v ii - v i v

C L2#LINE/L2 2708 AGAGATGATGGTGGCTTGGACCAGGGTGGTGGCAGTGGAGGTG 2666

Matrix = 25p53g.matrix

Kimura (with divCpGMod) = 42.61

Transitions / transversions = 1.11 (39/35)

Gap_init rate = 0.04 (9 / 228), avg. gap size = 1.56 (14 / 9)

[+](javascript:;) **250 33.3 43.7 0.0 MYEOV 1067 1297 (1836) +** [**L2a**](http://www.repeatmasker.org/cgi-bin/ViewRepeat?id=L2a)  **LINE/L2 2756 3089 (337) 2**

**ANNOTATION EVIDENCE:**

189 37.09 9.03 0.00 MYEOV 1067 1217 1879 + [**L2**](http://www.repeatmasker.org/cgi-bin/ViewRepeat?id=L2)  LINE/L2 2756 2921 161

189 37.09 9.03 0.00 MYEOV 1067 1217 (1879) L2#LINE/L2 2756 2921 (161) m_b1s601i1

MYEOV 1067 **CCTT--CTCCTCAGA**GTCCATTC-------AGGAGCAGGAAAGTTCATCT 1107

-- i ii?v i ------- v vi iv v v i

L2#LINE/L2 2756 **CCTTGCCCCCCTNCA**GTCTATTCTCAACACAGCAGCCAGAGTGATCCTTT 2805

MYEOV 1108 CAGACCCTAAATCCAGCCACGTCATGCCAC-GCTTAACACC-TCTAACAA 1155

i i v v i viii i iv v - i v - i iiii

L2#LINE/L2 2806 TAAAACATAAGTCAGATCATGTCACTCCTCTGCTCAAAACCCTCCAGTGG 2855

MYEOV 1156 **CTTCCCCTGGCACTTAGG**ACAGC-GTCTGGCTCCTTCCCTCGGCT--C**AT** 1202

v vv i iii iv- i viiv v vvi i-- v

L2#LINE/L2 **2856 CTTCCCATCTCACTCAGA**GTAAAAGCCAAAGTCCTTACAGTGGCCTAC**AA** 2905

MYEOV 1203 **G**GCCCT-CAGAATCTG 1217

- vi

L2#LINE/L2 2906 **G**GCCCTACATGATCTG 2921

Matrix = 25p53g.matrix

Kimura (with divCpGMod) = 52.53

Transitions / transversions = 1.39 (32/23)

Gap_init rate = 0.05 (7 / 150), avg. gap size = 2.14 (15 / 7)

250 26.51 6.74 0.00 MYEOV 1215 1297 1799 + [**L2a_3end**](http://www.repeatmasker.org/cgi-bin/ViewRepeat?id=L2a_3end) LINE/L2 92 180 337

250 26.51 6.74 0.00 MYEOV 1215 1297 (1799) L2a_3end#LINE/L2 92 180 (337) m_b1s601i2

MYEOV 1215 CTGCGTCACATACA**CCCCAG---CTCTCCCGAT---AGG**TCTCTGCACTC 1258

ivi v iv v --- v vi---i i i i

L2a_3end#LINE 92 CTGTTCCTCGAACA**CGCCAGGCACGCTCCCGCCTCAGGG**CCTTTGCACTT 141

MYEOV 1259 GCTGTTGCCTCTGCCTGGAACAGTCTCCCTCCTGGTGTC 1297

v iv i i v i i

L2a_3end#LINE 142 GCTGTTCCCTCTGCCTGGAACGCTCTTCCCCCAGATATC 180

Matrix = 25p53g.matrix

Kimura (with divCpGMod) = 29.88

Transitions / transversions = 1.44 (13/9)

Gap_init rate = 0.02 (2 / 82), avg. gap size = 3.00 (6 / 2)

[+](javascript:;) **27 5.5 2.6 0.0 MYEOV 2180 2217 (916) +** [**(CTC)n**](http://www.repeatmasker.org/cgi-bin/ViewRepeat?id=(CTC)n)  **Simple_repeat 1 39 (0) 3**

[+](javascript:;) **592 26.7 8.0 0.0 MYEOV 2218 2378 (755) C** [**MIR**](http://www.repeatmasker.org/cgi-bin/ViewRepeat?id=MIR)  **SINE/MIR (1) 261 87 4**

**ANNOTATION EVIDENCE:**

592 26.71 8.00 0.00 MYEOV 2218 2378 755 C [**MIR**](http://www.repeatmasker.org/cgi-bin/ViewRepeat?id=MIR)  SINE/MIR 87 261 1

592 26.71 8.00 0.00 MYEOV 2218 2378 (755) C MIR#SINE/MIR (1) 261 87 m_b1s601i3

MYEOV 2218 ATCATCATCCTCACTTGT**TGA**GGACGTCCTGTGTGCC------------A 2255

v v i vv i i vi v v i ------------

C MIR#SINE/MIR 261 ATAATAACCAACATTTAT**TGA**GCGCTTACTATGTGCCAGGCACTGTTCTA 212

MYEOV 2256 AGTGGTTTATATGCCCAGCCTCATTTAATCCTCAGAATGACTCCATGAGG 2305

i v i iviviv v ii i i

C MIR#SINE/MIR 211 AGCGCTTTACATGTATTAACTCATTTAATCCTCACAACAACCCTATGAGG 162

MYEOV 2306 TAGCTACTAAAACC--CCCCACTTAACAGATGAGGAAACTGAGGCCTAGA 2353

v vv ii-- i v vi

C MIR#SINE/MIR 161 TAGGTACTATTATTATCCCCATTTTACAGATGAGGAAACTGAGGCACAGA 112

MYEOV 2354 GAAGCTCAACAAGTTGCCTAAGTTC 2378

i i v ii v i v

C MIR#SINE/MIR 111 GAGGTTAAGTAACTTGCCCAAGGTC 87

Matrix = 25p53g.matrix

Kimura (with divCpGMod) = 31.66

Transitions / transversions = 1.15 (23/20)

Gap_init rate = 0.01 (2 / 160), avg. gap size = 7.00 (14 / 2)

[+](javascript:;) **216 27.7 3.0 0.0 MYEOV 2447 2511 (622) C** [**L2a**](http://www.repeatmasker.org/cgi-bin/ViewRepeat?id=L2a)  **LINE/L2 (1) 3425 3359 5**

[+](javascript:;) **288 29.6 5.0 0.9 MYEOV 2600 2715 (418) +** [**MER117**](http://www.repeatmasker.org/cgi-bin/ViewRepeat?id=MER117)  **DNA/hAT-Charlie 45 165 (32) 6**

[+](javascript:;) **230 20.3 12.3 3.0 MYEOV 2772 2870 (263) C** [**MIRb**](http://www.repeatmasker.org/cgi-bin/ViewRepeat?id=MIRb)  **SINE/MIR (38) 230 108 7**

[+](javascript:;) **248 26.8 1.3 2.1 MYEOV 2907 3053 (80) +** [**L4_A_Mam**](http://www.repeatmasker.org/cgi-bin/ViewRepeat?id=L4_A_Mam) **LINE/RTE-X 4690 4781 (209) 8**

**Supplementary Data Set 3**

LOCUS: NC_000011

REGION: 69295400..69295451 > GGTCTCTGCACTCGCTGTTGCCTCTGCCTGGAACAGTCTCCCTCCTGGTGTC

**Supplementary Data Set 4**

**(I)**

**RepeatMasker annotation:** “The query species was assumed to be **gorilla gorilla**

RepeatMasker version open-4.0.5, **sensitive mode**

Run with cross_match version 1.080812_RepBase Update 20140131, RM database version 20140131”.

**Author’s notes: contig CABD02065631, Region 2056..5186_ RepeatMasker Matrix used: Based on GC level query.**

**position in query- -position in repeat-**

**% % % query C matching repeat (left) end begin linkage**

**+ score div. del. ins. sequence begin end (left) + repeat class/family begin end (left) id/graphic**

[+](javascript:;) **421 36.2 3.1 0.0 CABD02065631 351 596 (2535) C** [**L2**](http://www.repeatmasker.org/cgi-bin/ViewRepeat?id=L2)  **LINE/L2 (511) 2908 2655 1**

[+](javascript:;) **215 30.6 9.2 3.0 CABD02065631 1143 1363 (1768) +** [**L2a**](http://www.repeatmasker.org/cgi-bin/ViewRepeat?id=L2a)  **LINE/L2 2932 3163 (224) 2**

**ANNOTATION EVIDENCE:**

215 31.33 5.06 3.23 CABD02065631 1143 1297 1834 + [**L2a_3end**](http://www.repeatmasker.org/cgi-bin/ViewRepeat?id=L2a_3end) LINE/L2 23 180 337

215 31.33 5.06 3.23 CABD02065631 1143 1297 (1834) L2a_3end#LINE/L2 23 180 (337) m_b1s601i1

CABD02065631 1143 ACACCTCTAACAACTTCCCCTGGCACTTAGGACAGCGTCTGGCTCCTTCC 1192

vi i vv v i iv ----- vv vi v vi

L2a_3end#LINE 23 ACCTCTCTGACCTCATCTCCTACCACT-----CTCCCCCTCGCTCACTCC 67

CABD02065631 1193 -CTC-AGCT-CG-TGGCCCTCAGAATCTGCGTCACACACACC---CTAGC 1235

- - i- i- ii vviv v v v iv i --- vv

L2a_3end#LINE 68 GCTCCAGCCACACTGGCCTCCTTGCTGTTCCTCGAACACGCCAGGCACGC 117

CABD02065631 1236 TCTCCCCATAGG-TCTCTGCACTCGCTGTTGCCTCTGCCTGGAACAGTCT 1284

i v vi -i i i v iv

L2a_3end#LINE 118 TCCCGCCTCAGGGCCTTTGCACTTGCTGTTCCCTCTGCCTGGAACGCTCT 167

CABD02065631 1285 CCCTCCTGGTGTC 1297

i i v i i

L2a_3end#LINE 168 TCCCCCAGATATC 180

Matrix = 25p53g.matrix

Kimura (with divCpGMod) = 37.81

Transitions / transversions = 0.96 (23/24)

Gap_init rate = 0.07 (11 / 154), avg. gap size = 1.18 (13 / 11)

184 30.22 11.47 2.80 CABD02065631 1221 1363 1768 + [**L2c_3end**](http://www.repeatmasker.org/cgi-bin/ViewRepeat?id=L2c_3end) LINE/L2 98 254 224

184 30.22 11.47 2.80 CABD02065631 1221 1363 (1768) L2c_3end#LINE/L2 98 254 (224) m_b1s601i2

CABD02065631 1221 CACACACACCCTAGCTCTCCCCA-----TAGGTCTCTGCACTCGCTGTTG 1265

v i vi i ii v-----ii? i i vi v

L2c_3end#LINE 98 CAAACACGCCGCAGTTCTTTCCTGCCTCCGNGCCTTTGCACATGCTGTTC 147

CABD02065631 1266 CCTCTGCCTGGAACAGTCT-CCCTCCTGGTGTCAT-TGTCT--CTGTGGT 1311

iiv - i vv vi i- v -- vivii

L2c_3end#LINE 148 CCTCTGCCTGGAATGCTCTTCCCCCCTCCTTCCACCTGGCTAACTCCTAC 197

CABD02065631 1312 GTGTCCTTC----CTGACCTT-----CCACCTCCACCAGGAGCCGACACT 1352

vii ---- v v i-----i v iv ----

L2c_3end#LINE 198 TCATCCTTCAAGNCTCAGCTCAGATGTCACCTCCTCCAGGAAGC----CT 243

CABD02065631 1353 TCCCCGACCCC 1363

i

L2c_3end#LINE 244 TCCCTGACCCC 254

Matrix = 25p53g.matrix

Kimura (with divCpGMod) = 36.37

Transitions / transversions = 1.41 (24/17)

Gap_init rate = 0.07 (10 / 142), avg. gap size = 2.20 (22 / 10)

[+](javascript:;) **27 9.1 4.3 0.0 CABD02065631 2179 2225 (906) +** [**(TCC)n**](http://www.repeatmasker.org/cgi-bin/ViewRepeat?id=(TCC)n)  **Simple_repeat 1 49 (0) 3**

[+](javascript:;) **593 26.2 7.5 0.7 CABD02065631 2226 2375 (756) C** [**MIRb**](http://www.repeatmasker.org/cgi-bin/ViewRepeat?id=MIRb)  **SINE/MIR (14) 254 94 4**

[+](javascript:;) **191 32.9 2.6 1.3 CABD02065631 2444 2520 (611) C** [**L2a**](http://www.repeatmasker.org/cgi-bin/ViewRepeat?id=L2a)  **LINE/L2 (1) 3425 3348 5**

[+](javascript:;) **238 32.5 5.3 0.8 CABD02065631 2599 2725 (406) +** [**MER117**](http://www.repeatmasker.org/cgi-bin/ViewRepeat?id=MER117)  **DNA/hAT-Charlie 46 178 (19) 6**

[+](javascript:;) **198 25.0 21.4 2.2 CABD02065631 2770 2859 (272) C** [**MIRb**](http://www.repeatmasker.org/cgi-bin/ViewRepeat?id=MIRb)  **SINE/MIR (38) 230 119 7**

[+](javascript:;) **279 26.4 1.3 2.1 CABD02065631 2905 3051 (80) +** [**L4_B_Mam**](http://www.repeatmasker.org/cgi-bin/ViewRepeat?id=L4_B_Mam) **LINE/RTE-X 4690 4781 (209) 8**

**(II)**

**RepeatMasker annotation:** “The query species was assumed to be **pongo abelii**

RepeatMasker version open-4.0.5, **sensitive mode**

Run with cross_match version 1.080812_RepBase Update 20140131, RM database version 20140131”.

**Author’s notes: contig ABGA01402731, Region 2369..5501_ RepeatMasker Matrix used: Based on GC level query.**

**position in query- -position in repeat-**

**% % % query C matching repeat (left) end begin linkage**

**+ score div. del. ins. sequence begin end (left) + repeat class/family begin end (left) id/graphic**

[+](javascript:;) **383 34.5 3.4 0.0 ABGA01402731 351 547 (2586) C** [**L2**](http://www.repeatmasker.org/cgi-bin/ViewRepeat?id=L2)  **LINE/L2 (511) 2908 2705 1**

[+](javascript:;) **244 30.8 36.8 5.4 ABGA01402731 1066 1342 (1791) +** [**L2a**](http://www.repeatmasker.org/cgi-bin/ViewRepeat?id=L2a)  **LINE/L2 2756 3120 (306) 2**

**ANNOTATION EVIDENCE:**

194 37.09 9.03 0.00 ABGA01402731 1066 1216 1917 + [**L2**](http://www.repeatmasker.org/cgi-bin/ViewRepeat?id=L2)  LINE/L2 2756 2921 161

194 37.09 9.03 0.00 ABGA01402731 1066 1216 (1917) L2#LINE/L2 2756 2921 (161) m_b1s601i1

ABGA01402731 1066 CCTT--CTCCTCAGAGTCCATTC-------AGGAGCAGGAAAGTTCATCT 1106

-- i ii?v i ------- v vi iv v v i

L2#LINE/L2 2756 CCTTGCCCCCCTNCAGTCTATTCTCAACACAGCAGCCAGAGTGATCCTTT 2805

ABGA01402731 1107 CAGACCCTAAGTCCAGCCACGTCATGCCAC-GCTTAACACC-TCTAACAA 1154

i i v v viii i iv v - i v - i iiii

L2#LINE/L2 2806 TAAAACATAAGTCAGATCATGTCACTCCTCTGCTCAAAACCCTCCAGTGG 2855

ABGA01402731 1155 CTTCCCCTGGCACTTAGGACAGC-GTCTAGCTCCTTCCCTCAGCT--CGT 1201

v vv i iii iv- i v iv v vvii i-- iv

L2#LINE/L2 2856 CTTCCCATCTCACTCAGAGTAAAAGCCAAAGTCCTTACAGTGGCCTACAA 2905

ABGA01402731 1202 GGCCCT-TAGGATCTG 1216

-i v

L2#LINE/L2 2906 GGCCCTACATGATCTG 2921

Matrix = 25p53g.matrix

Kimura (with divCpGMod) = 52.53

Transitions / transversions = 1.39 (32/23)

Gap_init rate = 0.05 (7 / 150), avg. gap size = 2.14 (15 / 7)

244 23.68 5.00 11.63 ABGA01402731 1214 1342 1791 + [**L2a_3end**](http://www.repeatmasker.org/cgi-bin/ViewRepeat?id=L2a_3end) LINE/L2 92 211 306

244 23.68 5.00 11.63 ABGA01402731 1214 1342 (1791) L2a_3end#LINE/L2 92 211 (306) m_b1s601i2

ABGA01402731 1214 CTGCGTCACACACACCCCAG---CTCTCCCGAT---GGGTCTCTGCACTC 1257

ivi v iv v --- v vi--- i i i

L2a_3end#LINE 92 CTGTTCCTCGAACACGCCAGGCACGCTCCCGCCTCAGGGCCTTTGCACTT 141

ABGA01402731 1258 GCTGTTGCCTCTGCCTGGAACAGTCTCCCTCCTGGTGTCATCGTCTCCAT 1307

v iv i i v i i -- ----

L2a_3end#LINE 142 GCTGTTCCCTCTGCCTGGAACGCTCTTCCCCCAGATATC--CG----CAT 185

ABGA01402731 1308 GGTGTGTCCTTCCTGACTTTCCACCTCCACCAGGT 1342

ivi -- i ------- vi

L2a_3end#LINE 186 GGCTCG--CTCCCT-------CACCTCCTTCAGGT 211

Matrix = 25p53g.matrix

Kimura (with divCpGMod) = 25.37

Transitions / transversions = 1.45 (16/11)

Gap_init rate = 0.13 (17 / 128), avg. gap size = 1.24 (21 / 17)

[+](javascript:;) **21 9.7 0.0 0.0 ABGA01402731 2186 2218 (915) +** [**(CTC)n**](http://www.repeatmasker.org/cgi-bin/ViewRepeat?id=(CTC)n)  **Simple_repeat 1 33 (0) 3**

[+](javascript:;) **608 26.1 8.0 0.0 ABGA01402731 2219 2379 (754) C** [**MIR**](http://www.repeatmasker.org/cgi-bin/ViewRepeat?id=MIR)  **SINE/MIR (1) 261 87 4**

[+](javascript:;) **229 33.8 2.4 0.0 ABGA01402731 2448 2548 (585) C** [**L2a**](http://www.repeatmasker.org/cgi-bin/ViewRepeat?id=L2a)  **LINE/L2 (1) 3425 3272 5**

[+](javascript:;) **306 28.3 5.2 0.8 ABGA01402731 2601 2728 (405) +** [**MER117**](http://www.repeatmasker.org/cgi-bin/ViewRepeat?id=MER117)  **DNA/hAT-Charlie 45 178 (19) 6**

[+](javascript:;) **232 23.6 10.4 2.4 ABGA01402731 2773 2871 (262) C** [**MIRb**](http://www.repeatmasker.org/cgi-bin/ViewRepeat?id=MIRb)  **SINE/MIR (38) 230 108 7**

[+](javascript:;) **271 30.1 0.0 2.0 ABGA01402731 2908 3007 (126) +** [**L4_B_Mam**](http://www.repeatmasker.org/cgi-bin/ViewRepeat?id=L4_B_Mam) **LINE/RTE-X 4690 4806 (184) 8**

**(III)**

**RepeatMasker annotation:** “The query species was assumed to be **macaca mulatta**

RepeatMasker version open-4.0.5, **sensitive mode**

Run with cross_match version 1.080812_RepBase Update 20140131, RM database version 20140131”.

**Author’s notes: contig AANU01213481, Region 1643..4737_ RepeatMasker Matrix used: Based on GC level query.**

**position in query- -position in repeat-**

**% % % query C matching repeat (left) end begin linkage**

**+ score div. del. ins. sequence begin end (left) + repeat class/family begin end (left) id/graphic**

[+](javascript:;) **403 36.6 3.1 0.0 AANU01213481 351 596 (2499) C** [**L2**](http://www.repeatmasker.org/cgi-bin/ViewRepeat?id=L2)  **LINE/L2 (511) 2908 2655 1**

[+](javascript:;) **253 26.1 2.6 9.7 AANU01213481 1188 1335 (1760) +** [**L2a**](http://www.repeatmasker.org/cgi-bin/ViewRepeat?id=L2a)  **LINE/L2 3005 3160 (215) 2**

**ANNOTATION EVIDENCE:**

253 25.74 0.00 11.40 AANU01213481 1188 1301 1794 + [**L2a_3end**](http://www.repeatmasker.org/cgi-bin/ViewRepeat?id=L2a_3end) LINE/L2 104 204 313

253 25.74 0.00 11.40 AANU01213481 1188 1301 (1794) L2a_3end#LINE/L2 104 204 (313) m_b1s601i1

AANU01213481 1188 CGCGTCACACACACCCCAGCTCTCGTGACGGGTCTCTGCACTCGCTGTTG 1237

i i vi i i v - i---- i i i v

L2a_3end#LINE 104 CACGCCAGGCACGCTCCCGC-CTCA----GGGCCTTTGCACTTGCTGTTC 148

AANU01213481 1238 CCTCCGCCTGGAACAGTCTTCCTCCTGGTGTCATCATCTCCGTGGTGCGT 1287

i iv i v i ------ iv i iv -

L2a_3end#LINE 149 CCTCTGCCTGGAACGCTCTTCCCCCAGAT------ATCCGCATGGCTCG- 191

AANU01213481 1288 CCTTCCTGACCTTC 1301

- i v i

L2a_3end#LINE 192 -CTCCCTCACCTCC 204

Matrix = 25p53g.matrix

Kimura (with divCpGMod) = 28.39

Transitions / transversions = 2.25 (18/8)

Gap_init rate = 0.12 (13 / 113), avg. gap size = 1.00 (13 / 13)

194 27.54 11.54 4.17 AANU01213481 1192 1335 1760 + [**L2b_3end**](http://www.repeatmasker.org/cgi-bin/ViewRepeat?id=L2b_3end) LINE/L2 96 251 215

194 27.54 11.54 4.17 AANU01213481 1192 1335 (1760) L2b_3end#LINE/L2 96 251 (215) m_b1s601i2

AANU01213481 1192 TCACACACACC---CCAGCTCTCGTGAC-GGGTCTCTGCACTCGCTGTTG 1237

iv i --- iv i ivv - i i ?i v

L2b_3end#LINE 96 TCGAACACGCCAGGCTCGCTCCCGCCTCAGGGCCTTTGCACNTGCTGTTC 145

AANU01213481 1238 CCTCCGCCTGGAACAGTCTTCC-----TCCTGGTGTCATCATCTCCGTGG 1282

i ivi ----- i v iv vii v - iv

L2b_3end#LINE 146 CCTCTGCCTGGAACGCCCTTCCCCACCTCTTCGCCTGGCCAACTCC-TAC 194

AANU01213481 1283 TGCGTCCTTC----CTGACCTT-----CCACCTCCACCAGGTGCCAACAC 1323

- i ---- v v i-----i v ---- vv

L2b_3end#LINE 195 T-CATCCTTCAGGTCTCAGCTCAAATGTCACCTCCTCCAGG----AAGCC 239

AANU01213481 1324 TTCCCCGACCCC 1335

i i

L2b_3end#LINE 240 CTCCCTGACCCC 251

Matrix = 25p53g.matrix

Kimura (with divCpGMod) = 30.87

Transitions / transversions = 1.31 (21/16)

Gap_init rate = 0.08 (11 / 143), avg. gap size = 2.18 (24 / 11)

[+](javascript:;) **23 11.4 0.0 0.0 AANU01213481 2137 2174 (921) +** [**(TCC)n**](http://www.repeatmasker.org/cgi-bin/ViewRepeat?id=(TCC)n)  **Simple_repeat 1 38 (0) 3**

[+](javascript:;) **584 27.0 7.6 0.0 AANU01213481 2175 2333 (762) C** [**MIR**](http://www.repeatmasker.org/cgi-bin/ViewRepeat?id=MIR)  **SINE/MIR (4) 258 87 4**

[+](javascript:;) **218 31.4 4.8 10.6 AANU01213481 2402 2533 (562) C** [**L2b**](http://www.repeatmasker.org/cgi-bin/ViewRepeat?id=L2b)  **LINE/L2 (1) 3386 3263 5**

[+](javascript:;) **317 29.7 4.5 0.0 AANU01213481 2555 2682 (413) +** [**MER117**](http://www.repeatmasker.org/cgi-bin/ViewRepeat?id=MER117)  **DNA/hAT-Charlie 45 178 (19) 6**

**(IV)**

**RepeatMasker annotation:** “The query species was assumed to be **ceratotherium simum simum**

RepeatMasker version open-4.0.5, **sensitive mode**

Run with cross_match version 1.080812_RepBase Update 20140131, RM database version 20140131”.

**Author’s notes: contig AKZM01047399, Region (c) 67309..70265_ RepeatMasker Matrix used: Based on GC level query.**

**position in query- -position in repeat-**

**% % % query C matching repeat (left) end begin linkage**

**+ score div. del. ins. sequence begin end (left) + repeat class/family begin end (left) id/graphic**

[+](javascript:;) **411 32.1 3.0 0.0 AKZM01047399 37 167 (2790) C** [**L3**](http://www.repeatmasker.org/cgi-bin/ViewRepeat?id=L3)  **LINE/CR1 (135) 3964 3830 1**

[+](javascript:;) **434 32.0 7.5 2.3 AKZM01047399 330 894 (2063) C** [**L2b**](http://www.repeatmasker.org/cgi-bin/ViewRepeat?id=L2b)  **LINE/L2 (109) 3266 2665 2**

[+](javascript:;) **285 28.3 10.0 2.9 AKZM01047399 1426 1780 (1177) +** [**L2b**](http://www.repeatmasker.org/cgi-bin/ViewRepeat?id=L2b)  **LINE/L2 2792 3196 (179) 3**

**ANNOTATION EVIDENCE:**

274 28.39 18.34 2.88 AKZM01047399 1426 1668 1289 + [**L2**](http://www.repeatmasker.org/cgi-bin/ViewRepeat?id=L2)  LINE/L2 2792 3080 2

274 28.39 18.34 2.88 AKZM01047399 1426 1668 (1289) L2#LINE/L2 2792 3080 (2) m_b1s601i3

AKZM01047399 1426 CAGAATGCTCCTCTCGGGCCGTAAATCAAGCCATGTC--TCCGCATGCTC 1473

i v i iiiiv i i iii -- v -

L2#LINE/L2 2792 CAGAGTGATCCTTTTAAAACATAAGTCAGATCATGTCACTCCTC-TGCTC 2840

AKZM01047399 1474 ATGGCC-TCCAGCGACTTCCCCTGGCACTTAGGATGAT-------GTC-- 1513

vii - i i v vv i ii i v------- --

L2#LINE/L2 2841 AAAACCCTCCAGTGGCTTCCCATCTCACTCAGAGTAAAAGCCAAAGTCCT 2890

AKZM01047399 1514 -----TGCTCCACGAGGCCCTGGA------GGTCC-----AGCCTGCCTG 1547

----- vi i i iv ------ -----vi vi

L2#LINE/L2 2891 TACAGTGGCCTACAAGGCCCTACATGATCTGGTCCCCCGTTACCTCTCTG 2940

AKZM01047399 1548 GCTCCGTC-------------CTGCTCACACACTG---TCC--CCACCTT 1579

i ii i ------------- iv i v v--- -- vi

L2#LINE/L2 2941 ACCTCATCTCCTACCACTCTCCCCCTCGCTCACTCCGCTCCAGCCACACT 2990

AKZM01047399 1580 GGCCTCCAGG----TCCT---GCAGGCTTGGCTCCCTGAGCTCTCCTCAC 1622

vv ---- ---i v iv v v --- iiv -

L2#LINE/L2 2991 GGCCTCCTTGCTGTTCCTCGAACACGCCAGGCACGCT---CCTGCCTCA- 3036

AKZM01047399 1623 CTGGGCCTTTGTGCATGCTGTTCCCTCTGCCCAGAACGGCCTCCCC 1668

-- ii v ii vi i

L2#LINE/L2 3037 --GGGCCTTTGCACTTGCTGTTCCCTCTGCCTGGAACGCTCTTCCC 3080

Matrix = 25p53g.matrix

Kimura (with divCpGMod) = 36.23

Transitions / transversions = 1.68 (42/25)

Gap_init rate = 0.08 (19 / 242), avg. gap size = 3.16 (60 / 19)

285 28.21 6.02 2.90 AKZM01047399 1540 1780 1177 + [**L2b_3end**](http://www.repeatmasker.org/cgi-bin/ViewRepeat?id=L2b_3end) LINE/L2 39 287 179

285 28.21 6.02 2.90 AKZM01047399 1540 1780 (1177) L2b_3end#LINE/L2 39 287 (179) m_b1s601i4

AKZM01047399 1540 CCTGCCTGGCTCCGTCCTGCTCACAC-ACTGTCCCCACCTTGGCCTCCAG 1588

i vvv -i i v -i vivv vi vv

L2b_3end#LINE 39 CCTACCACTCTCC-CCTTGCTCACTCTGCTCCAGCCACACTGGCCTCCTT 87

AKZM01047399 1589 G----TCCT---GCAGGCTTGGCTCCCTGAGCTCTCCTCACCTGGGCCTT 1631

---- ---i v iv v --- i v ---

L2b_3end#LINE 88 GCTGTTCCTCGAACACGCCAGGCTCGCT---CCCGCCTCA---GGGCCTT 131

AKZM01047399 1632 TGTGCATGCTGTTCCCTCTGCCCAGAACGGCCT-CCCCGCCT-TTCCCCT 1679

ii ? ii v - i - v

L2b_3end#LINE 132 TGCACNTGCTGTTCCCTCTGCCTGGAACGCCCTTCCCCACCTCTTCGCCT 181

AKZM01047399 1680 GGC-GGCTCCTTCTT-TCCTTTGGTTGTCAGTTTCCAGGCCACGTGCTC- 1726

-ii v i- ii v v i ivv v i v v -

L2b_3end#LINE 182 GGCCAACTCCTACTCATCCTTCAGGTCTCAGCTCAAATGTCACCTCCTCC 231

AKZM01047399 1727 AGTGCGCCCTTGCTGACCACCCCGCCAGTGGCTGCGGCCC--CTCTGACT 1774

viv iv v v v v v v v vv -- ivi

L2b_3end#LINE 232 AGGAAGCCCTCCCTGACCCCCCAGGCCGGGTCAGGCGCCCTCCTCTGGGC 281

AKZM01047399 1775 CTGCCC 1780

iv

L2b_3end#LINE 282 CCCCCC 287

Matrix = 25p53g.matrix

Kimura (with divCpGMod) = 34.98

Transitions / transversions = 0.71 (27/38)

Gap_init rate = 0.07 (16 / 240), avg. gap size = 1.38 (22 / 16)

[+](javascript:;) **473 28.5 0.8 0.8 AKZM01047399 2690 2820 (137) C** [**MIRb**](http://www.repeatmasker.org/cgi-bin/ViewRepeat?id=MIRb)  **SINE/MIR (46) 222 92 4**

[+](javascript:;) **23 17.0 5.7 2.8 AKZM01047399 2823 2892 (65) +** [**(CTCC)n**](http://www.repeatmasker.org/cgi-bin/ViewRepeat?id=(CTCC)n)  **Simple_repeat 1 72 (0) 5**

**(V)**

**RepeatMasker annotation:** “The query species was assumed to be **leptonychotes weddellii**

RepeatMasker version open-4.0.5, **sensitive mode**

Run with cross_match version 1.080812_RepBase Update 20140131, RM database version 20140131”.

**Author’s notes: contig APMU01114670, Region (c) 1166..3026_RepeatMasker Matrix used: Based on GC level query.**

**position in query- -position in repeat-**

**% % % query C matching repeat (left) end begin linkage**

**+ score div. del. ins. sequence begin end (left) + repeat class/family begin end (left) id/graphic**

[+](javascript:;) **362 30.1 7.3 3.7 APMU01114670 649 976 (885) +** [**L2b**](http://www.repeatmasker.org/cgi-bin/ViewRepeat?id=L2b)  **LINE/L2 2962 3302 (73) 1**

**ANNOTATION EVIDENCE:**

362 30.06 7.33 3.66 APMU01114670 649 976 885 + [**L2b_3end**](http://www.repeatmasker.org/cgi-bin/ViewRepeat?id=L2b_3end) LINE/L2 53 393 73

362 30.06 7.33 3.66 APMU01114670 649 976 (885) L2b_3end#LINE/L2 53 393 (73) m_b1s601i0

APMU01114670 649 CCTGCTCACTCAAGGTTCCCATCCC--TGTCCCCAGGCCCTGCTGGTCC- 695

i --v i vii v -- v i ---- i v -

L2b_3end#LINE 53 CTTGCTCACTC--TGCTCCAGCCACACTGGCCTC----CTTGCTGTTCCT 96

APMU01114670 696 --AGCTCCCTGAGCTCTCCCCAACCT--GTGCCTCTGTGCATGCTGTTCC 741

-- i v v iii v i vi -- v i ii ?

L2b_3end#LINE 97 CGAACACGCCAGGCTCGCTCCCGCCTCAGGGCCTTTGCACNTGCTGTTCC 146

APMU01114670 742 CTCTGCCTGGAACAGTCCCCCTCCCCCTC----CC---CCCACACCTTCT 784

- i ii i v ---- --- v v v

L2b_3end#LINE 147 CTCTGCCTGGAAC-GCCCTTCCCCACCTCTTCGCCTGGCCAACTCCTACT 195

APMU01114670 785 TG-CCTTCGGTTCTCAGTTTCAAGGTCACCTCCTC-AGCATGGCCTTGCT 832

ii- i v i iv v - v v v iv

L2b_3end#LINE 196 CATCCTTCAGGTCTCAGCTCAAATGTCACCTCCTCCAGGAAGCCCTCCCT 245

APMU01114670 833 GACCACCTCG-CTGAG-CAGCCGCCC-CATGTCTGCCTCCTCTGT--GGC 877

v iv - i i - v - v v vv i i v --vi

L2b_3end#LINE 246 GACCCCCCAGGCCGGGTCAGGCGCCCTCCTCTGGGCCCCCCCAGTCCTAC 295

APMU01114670 878 CCTGCACAC---AAGCTGTTGACGTTTGGGATTGTCATTGTGTGTCTTCT 924

- ---ii i i ?ivi i ?-- i- - v i i

L2b_3end#LINE 296 CCTGC-CACTCTGGGTTATNATTGTCTGN--TTA-CAT-GTCTGTCTCCC 340

APMU01114670 925 TGTCTAGTCCCTGTCCCCCTTGAGCACAGAGACTGG-CCTCTCTTGTCCA 973

ivv i v iv vv i v vi i i -i v i

L2b_3end#LINE 341 CCACTGGACTGTGAGCTCCGTGAGGGCAGGGACCGGGTCTGTCTTGTTCA 390

APMU01114670 974 CTG 976

i

L2b_3end#LINE 391 CCG 393

Matrix = 25p53g.matrix

Kimura (with divCpGMod) = 36.04

Transitions / transversions = 1.24 (51/41)

Gap_init rate = 0.08 (26 / 327), avg. gap size = 1.42 (37 / 26)

[+](javascript:;) **282 21.9 0.0 2.7 APMU01114670 1274 1348 (513) +** [**Tigger12**](http://www.repeatmasker.org/cgi-bin/ViewRepeat?id=Tigger12) **DNA/TcMar-Tigger 538 610 (1865) 2**

[+](javascript:;) **361 31.7 3.2 0.0 APMU01114670 1631 1750 (111) C** [**MIR**](http://www.repeatmasker.org/cgi-bin/ViewRepeat?id=MIR)  **SINE/MIR (49) 213 90 3**

**(VI)**

**RepeatMasker annotation:** “The query species was assumed to be **dasypus novemcinctus**

RepeatMasker version open-4.0.5, **sensitive mode**

Run with cross_match version 1.080812_RepBase Update 20140131, RM database version 20140131”.

**Author’s notes: contig AAGV03306376, Region (c) 17932..19916_RepeatMasker Matrix used: Based on GC level query.**

**position in query- -position in repeat-**

**% % % query C matching repeat (left) end begin linkage**

**+ score div. del. ins. sequence begin end (left) + repeat class/fami begin end (left) id/graphic**

[+](javascript:;) **381 26.7 4.0 1.1 AAGV03306376 805 999 (986) +** [**L2b**](http://www.repeatmasker.org/cgi-bin/ViewRepeat?id=L2b)  **LINE/L2 2970 3164 (262) 1**

**ANNOTATION EVIDENCE:**

299 26.88 4.62 4.62 AAGV03306376 805 999 986 + [**L2a_3end**](http://www.repeatmasker.org/cgi-bin/ViewRepeat?id=L2a_3end) LINE/L2 61 255 262

299 26.88 4.62 4.62 AAGV03306376 805 999 (986) L2a_3end#LINE/L2 61 255 (262) m_b1s601i0

AAGV03306376 805 TCACGCCGTCCCCAGCCACACCCTCCCACCCTGAGTTCTGCTGTCCCTGC 854

v - i ivv i-- ------ i vv

L2a_3end#LINE 61 TCACTCCG-CTCCAGCCACACTGGCCT--CCT------TGCTGTTCCTCG 101

AAGV03306376 855 AACACCCCAAG-ATCCCCACGCCTCGGGGCCTTTGCACATGCTGATCCCC 903

v i - iv i v i v v i

L2a_3end#LINE 102 AACACGCCAGGCACGCTCCCGCCTCAGGGCCTTTGCACTTGCTGTTCCCT 151

AAGV03306376 904 C-GCCTGGAGCAGTCTCCCCC--GCTCTCCTCTCGGCCGGCGCCTTC-TC 949

- i iv i -- v v v vi iv v i -i

L2a_3end#LINE 152 CTGCCTGGAACGCTCTTCCCCCAGATATCCGCATGGCTCGCTCCCTCACC 201

AAGV03306376 950 TCCGGCTGCTCTCAGTTTCGGCGTCACCTCGCTAGTG--GCCTGC--TGA 995

vv v v iv i iviii ivii -- v --

L2a_3end#LINE 202 TCCTTCAGGTCTTTGCTCAAATGTCACCTTCTCAGTGAGGCCTTCCCTGA 251

AAGV03306376 996 CCAC 999

L2a_3end#LINE 252 CCAC 255

Matrix = 25p53g.matrix

Kimura (with divCpGMod) = 32.04

Transitions / transversions = 1.00 (25/25)

Gap_init rate = 0.08 (15 / 194), avg. gap size = 1.20 (18 / 15)

381 26.67 3.85 0.00 AAGV03306376 830 979 1006 + [**L2b_3end**](http://www.repeatmasker.org/cgi-bin/ViewRepeat?id=L2b_3end) LINE/L2 72 227 239

381 26.67 3.85 0.00 AAGV03306376 830 979 (1006) L2b_3end#LINE/L2 72 227 (239) m_b1s601i1

AAGV03306376 830 CCACCCTGAGTTC--TGCTGTCCCTGCAACACCCCAAGATC-CCCACGCC 876

v ivi -- i vv v i v - i v

L2b_3end#LINE 72 CCACACTGGCCTCCTTGCTGTTCCTCGAACACGCCAGGCTCGCTCCCGCC 121

AAGV03306376 877 TCGGGGCCTTTGCACATGCTGATCCCCC-GCCTGGAGCAGTCTCCCCCGC 925

i ? v i - i ivi i i

L2b_3end#LINE 122 TCAGGGCCTTTGCACNTGCTGTTCCCTCTGCCTGGAACGCCCTTCCCCAC 171

AAGV03306376 926 -TCTCCTCTCGGCCGGCGCCTTCTC-TCCGGCTGCTCTCAGTTTCGGCGT 973

- i v ii ii v v - vv v v i iviii

L2b_3end#LINE 172 CTCTTCGCCTGGCCAACTCCTACTCATCCTTCAGGTCTCAGCTCAAATGT 221

AAGV03306376 974 CACCTC 979

L2b_3end#LINE 222 CACCTC 227

Matrix = 25p53g.matrix

Kimura (with divCpGMod) = 31.79

Transitions / transversions = 1.29 (22/17)

Gap_init rate = 0.03 (5 / 149), avg. gap size = 1.20 (6 / 5)
